# Molecular studies into cell biological role of Copine-4 in Retinal Ganglion Cells

**DOI:** 10.1371/journal.pone.0255860

**Published:** 2021-11-30

**Authors:** Manvi Goel, Angel M. Aponte, Graeme Wistow, Tudor C. Badea

**Affiliations:** 1 Retinal Circuit Development & Genetics Unit, Neurobiology Neurodegeneration & Repair Laboratory, NEI, National Institutes of Health, Bethesda, Maryland, United States of America; 2 Department of Neuroscience, College of Medicine, The Ohio State University, Columbus, Ohio, United States of America; 3 Proteomics Core, NHLBI, National Institutes of Health, Bethesda, Maryland, United States of America; 4 Section on Molecular Structure and Functional Genomics, NEI, National Institutes of Health, Bethesda, Maryland, United States of America; 5 Faculty of Medicine, Research and Development Institute, Transilvania University of Brasov, Brasov, Romania; Instituto Murciano de Investigacion y Desarrollo Agrario y Alimentario, SPAIN

## Abstract

The molecular mechanisms underlying morphological diversity in retinal cell types are poorly understood. We have previously reported that several members of the Copine family of Ca-dependent membrane adaptors are expressed in Retinal Ganglion Cells and transcriptionally regulated by Brn3 transcription factors. Several Copines are enriched in the retina and their over-expression leads to morphological changes -formation of elongated processes-, reminiscent of neurites, in HEK293 cells. However, the role of Copines in the retina is largely unknown. We now investigate Cpne4, a Copine whose expression is restricted to Retinal Ganglion Cells. Over-expression of Cpne4 in RGCs in vivo led to formation of large varicosities on the dendrites but did not otherwise visibly affect dendrite or axon formation. Protein interactions studies using yeast two hybrid analysis from whole retina cDNA revealed two Cpne4 interacting proteins–Host Cell Factor 1 and Morn2. Mass Spectrometry analysis of retina lysate pulled down using Cpne4 or its vonWillebrand A domain showed 207 interacting proteins. A Gene Ontology analysis of the discovered proteins suggests that Cpne4 is involved in several metabolic and signaling pathways in the retina.

## 1. Introduction

Retinal Ganglion Cells (RGCs) in the retina transmit visual signals received by photoreceptors to the brain for processing visual inputs. Different RGC sub-types are responsible for computing different aspects of the visual stimuli. The combinatorial expression of transcription factors in different RGC sub-types regulates cell specific morphologies and physiology by controlling molecules involved in the development of dendrite/axon morphology, synapse formation and function [1–10].

How cell-specific morphologies develop in the retina is not well understood. One likely mechanism is that transcription factors encode specific morphological features via adhesion molecules or cytoskeletal elements they regulate. For example, Tbr1 regulates cell adhesion molecules Cdh8 and Sorcs3 resulting in dendritic stratification of JamB^+^ RGCs in the Off sublamina [11]. Other such molecules might be responsible for cell specific morphologies in other RGC types. Copines are a family of such candidate cell morphology determinants, regulated by Brn3 transcription factors. Copines consist of two C2 domains (C2A and C2B) and a vonWillebrand A (vWA) domain [12,13]. The Copine C2 domains are similar to those found in a variety of vesicular traffic proteins such as Synaptotagmins, Munc18, Rabphilin3A and Doc2, and have been involved in calcium dependent binding to cell membranes [14]. vWA domains are typically found extracellularly in several proteins (e.g., integrins) and are involved in protein-protein interactions [15]. However, Copines (and their vWA domain) are intracellular proteins that interact transiently with the inner leaflet of the plasma membrane.

There are nine Copines in mammals- Cpne1- 9 [12,16–18]. Of these, Cpne1, 2 and 3 are expressed ubiquitously. Cpne4, 5, 6, 7, 8 and 9 are enriched in neurons. Cpne5 and 8 are also expressed in other tissues such as kidneys, lungs, testes and mammary glands [19,20].

Copines are conserved across several species. They have previously been shown to be important in a variety of cellular functions. Copines are involved in myofilament stability in C. elegans and plant growth in Arabidopsis [21,22]. Copines are also involved in cytokinesis and contractile vacuole function by regulating cAMP signaling in Dictyostelium[23]. Copine A has also been shown to interact with actin filaments to regulate chemotaxis and adhesion in Dictyostelium[24].

In the nervous system, Copines were first seen to be localized in hippocampus and olfactory bulb neurons in mouse brain [18,25]. Cpne6 is expressed in the hippocampus and is required for regulating the spine morphology during long term potentiation in hippocampus by regulating the Rac-LIMK-Cofilin and BDNF-TrkB pathways [26,27]. Cpne1 has been previously seen to be upregulated during development and is required for hippocampal progenitor cell differentiation into neurons [28,29]. Cpne7 is expressed in sublaterodorsal nucleus in pontine segmental area and is required for rapid eye movement (REM) sleep [30].

In the retina, we have previously reported that Cpne4, 5, 6 and 9 are enriched in the inner retina and they are regulated by Brn3b and Brn3a [10,31]. Whereas Cpne5, 6 and 9 were expressed in most of the Ganglion Cell Layer (GCL) as well as the Inner Nucelar Layer (INL), Cpne4 is the only Copine specifically expressed in RGCs (with the exception of one INL amacrine cell type) [31]. Using over-expression studies in HEK293 cells, we found that Copines can significantly alter cell morphology, inducing elongated membrane processes. A previous study had investigated the potential interacting proteins for Cpne1, 2 and 4 using yeast two hybrid (Y2H) analysis on a mouse embryonic cDNA library [32]. In the current study, we further explore the effects of Cpne4 expression in RGCs and study its protein interactions using a Y2H analysis using retina cDNA library and Mass Spectrometry on total retina lysate, to identify its cell biological functions and role in RGCs.

## 2. Materials and methods

### 2.1 Transfection in HEK293 cells

**Fig 1:**
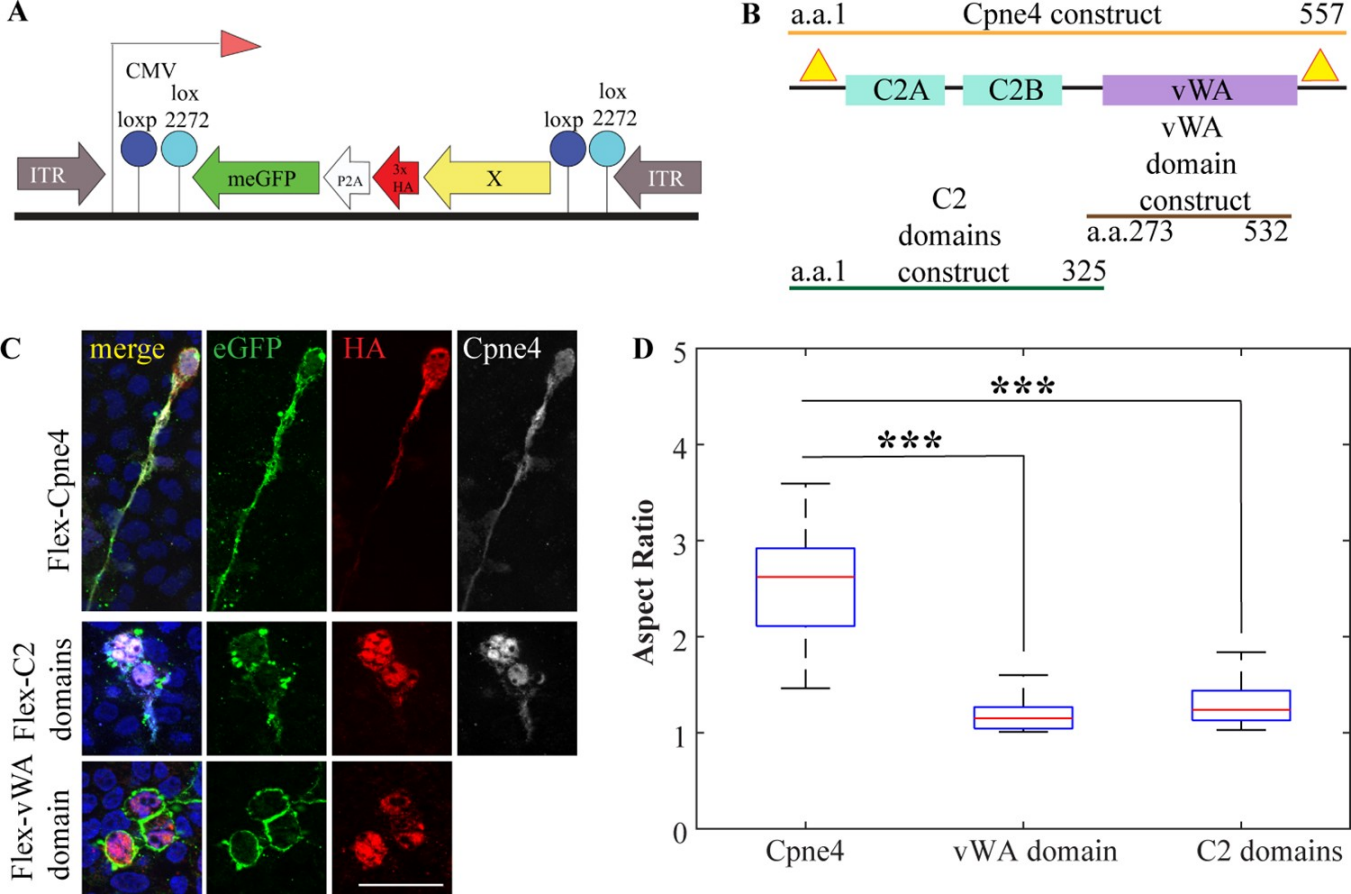
Cpne4 and Cpne4 dominant negative transfections in HEK293. (A) Map of the Flex construct. (B) Map of domain structure of Cpne4 shows three domains of the Cpne4 protein- two C2 domains (*blue*) and a vWA domain (*purple*). The location of the three Cpne4 plasmid constructs are shown for full length Cpne4 construct (*orange*), vWA domain construct (*brown*) and C2 domains construct (*green*). The binding of the two Cpne4 antibodies- N-terminal and C-terminal antibodies on the Cpne4 protein are indicated by yellow triangles. (C) Representative images of HEK293 cells transfected with expression constructs for full length Cpne4 (top row), C2 domains construct (middle row) and vWA domain construct (bottom row). The cells were counterstained for eGFP (*green*), HA (*red*) and N-terminal or C-terminal Cpne4 (*white*) antibodies and nuclear marker DAPI (*blue*). (D) Morphometric analysis showing aspect ratios of an ellipse fitted to the cells calculated for Cpne4, vWA domain and C2 domains transfected cells. The boxes show interquartile intervals with the median indicated with a red line and the whiskers represent the range of the observations. The mean and median aspect ratios for each group and p-values of comparisons of vWA domain and C2 domains transfected cells to Cpne4 transfected cells are given in Table 2.***, p< 0.001. Scale bar: 50μm.

The cDNA for full length Cpne4, two C2 domain or vWA domain were cloned into pAAV-FLEX-HA-T2A-meGFP plasmid vector (Fig 1A). The cDNA is in frame with 3XHA (3 tandem copies of Hemagglutinin or HA antigen tag- 5’TACCCATACGATGTTCCAGATTACGCT 3’ or 5’TATCCATATGATGTTCCAGATTATGCT 3’) and separated from membrane enhanced green fluorescent protein (meGFP) by a P2A peptide sequence. The constructs were transfected into human embryonic kidney 293 cells expressing Cre (HEK293^*Cre*^*)* using Lipofectamine (Invitrogen, Carlsbad, CA). The transfected cells were fixed in 2% paraformaldehyde (PFA) after 48 hours and processed for immunofluorescence as described in section 2.4.

### 2.2 Mouse lines

Adult *Brn3b*^*KO/KO*^ (or Brn3b KO: Brn3b knockout) [33] and *Brn3b*^*W/WT*^ (or Brn3b WT or WT: wild-type) littermates were used for immunohistochemistry (IHC). Postnatal day 0 (P0) or P14 *Brn3b*^*Cre/WT*^ mice were used for AAV1 virus infections. Adult wild-type (C57/Bl6 –SV129 mixed background) mice were used for retina pulldowns and mass spectrometry experiments, to determine the protein interactors of Cpne4. All animal procedures were approved by the National Eye Institute (NEI) Animal Care and Use Committee under protocol NEI640.

### 2.3 Virus infections in retina

Flex-meGFP-P2A-HA-Cpne4, described in section 2.1, was packaged into adeno-associated virus 1 (AAV1, henceforth AAV1-Cpne4). Postnatal 0 (P0) or P-15 Brn3b^Cre/WT^ mice were used for these experiments. P0 pups were anesthetized on ice for 30 seconds and a slit was cut in the eye lid. Intraocular injections of 0.5 ìl (1e9 viral particles/ìl) of AAV1-Cpne4 or AAV1-eGFP control virus were done in the eyes using pulled glass capillaries fitted onto a Femtojet device (Eppendorf, Enfield, CT), as previously described [1]. Injections were aimed at the scleral region adjacent to the limbus. P-15 mice were anesthetized with 100 mg/kg ketamine and 10 mg/kg xylazine before intraocular injections. Both P0 and P-15 pups were returned to their mothers after the experiments. Intraocular injections do not have systemic effects, and the injections in pups heal rapidly. If necessary, the corneal surfaces were flushed with a dilute betadine-saline solution and triple ophthalmic ointment was applied after the procedure. Animals were monitored daily by the investigators and the facility care staff for up to 5 days for signs of eye infection. General health of the mice was observed daily until euthanasia.

The mice were euthanized and eyes were collected at 2–3 months of age (adult animals) and flat-mounted for immunostaining. The eyes were fixed in 4% paraformaldehyde for 15 minutes and retinas were dissected to make a flat mount preparation. The retinas were again fixed for 30 minutes and then washed three times with phosphate buffered saline+ 0.5% Triton-X 100 (PBST). Immunofluorescence was performed as described below. Total number of animals used (1 retina was used per animal) for this experiment is given in section 3.2.

### 2.4 Immunofluorescence

Brn3b WT and KO sections were co-immunostained to confirm the presence of different interactor proteins identified from Y2H and mass spectrometry analysis in the retinal ganglion cells. Similar process was followed for immunofluorescence of transfected HEK293 cells on coverslips. The sections or cells were incubated with blocking solution- 10% bovine serum albumin (BSA), 10% normal donkey serum (NDS) and 0.5% Triton X 100, for one hour at room temperature. The blocking solution was then replaced with primary antibody solution containing the antibodies at the required concentrations and incubated overnight, at 4°C. Sections or cells were washed three times with PBST and incubated with the secondary antibody solutions for one hour at room temperature. The sections or cells were washed again with PBST and coverslipped.

For staining the AAV1 infected retina flatmounts, the retinas were incubated in blocking solution, overnight at 4°C. The blocking solution was then replaced with primary antibody solution and retinas incubated for 48 hours at 4°C. The retinas were then washed three times in PBST, secondary antibody solution was added, and retinas were incubated overnight at 4°C. The retinas were washed again three times with PBST, carefully mounted on glass slides and coverslips were placed. Image acquisition was on either a Axioimager Z2 fitted with an apotome device, or on a LSM 880 confocal microscope (both from Zeiss, White Plains, NY). The images were taken as 1 ìm thick z-stacks, and the images were stacked using ImageJ. Colocalization analysis of Copine4-interacting proteins in HEK293 cells was performed using the “Coloc 2” plugin in ImageJ.

All details of primary antibodies used are given in Table 1.

**Table 1:** List of antibodies used for immunofluorescence and WB.

Antigen	immunogen	Antibody details	Dilution used for IHC (and WB)
C- terminal Cpne4	EVYESSRTLA	made in house, rabbit polyclonal	1:2000 (1:1000 for WB)
N- terminal Cpne4	KKMSNIYESAANTLGIFNS	made in house, rabbit polyclonal	1:400 (1:1000 for WB)
Brn3a	Human Brn3a a.a.186–224 protein 10 fusion (pGEMEX)	Millipore, mouse monoclonal, Cat# MAB1585	1:20
Brn3b	Human Brn3b aa184–252 GST fusion	made in house, rabbit polyclonal	1:20
mouse HA	CYPYDVPDYASL	Covance, mouse monoclonal, clone 16B12	1:100
rabbit HA	YPYDVPDYASL	Cell Signaling Technologies, rabbit polyclonal, Cat# 3724	1:100
GFP	Recombinant full length GFP	Abcam, chicken polyclonal, Cat# ab13970	1:700
Morn2	Recombinant protein against the epitope- MNGFGRLEHFSGAVYEGQFK DNMFHGLGTYTFPNGAKYTG NFNENRVEGEGEYTDIQGLEWS GNFHFTAAPDLKLK	Sigma prestige antibodies, rabbit polyclonal, Cat# HPA057815	1:100
HCF1	aa 206–251 mapping near the N-terminus of human HCF1	Santa Cruz Biotechnology, mouse monoclonal, Cat# sc-390950	1:50
Map1b	aa 6–31 at the N-terminus of human MAP-1B	Santa Cruz Biotechnology, mouse monoclonal, Cat# sc-365668	1:50 (1:250)
Pan 14-3-3	aa 1–30 at the N-terminus of human pan 14-3-3	Santa Cruz Biotechnology, mouse monoclonal, Cat# sc-1657	1:50 (1:500)
SV2	Purified synaptic vesicles	Developmental Studies Hybridoma Bank, DSHB, of the University of Iowa, USA, mouse monoclonal	1:500 (1:2000)
Syntaxin1	Recombinant protein corresponding to AA 1 to 262 from rat Syntaxin1A (UniProt Id: P32851)	Synaptic systems, mouse monoclonal, Cat# 110011	1:200 (1:2500)
GST	26 kDa GST specific domain of a fusion protein encoded by a pGEX.3X recombinant vector	Santa Cruz Biotechnology, rabbit polyclonal, Cat# sc-459	(1:750)

### 2.5 Yeast two hybrid (Y2H) analysis

A Gal4 based Y2H analysis was performed to identify the proteins that interact with Cpne4 vWA domain. An adult mouse retina cDNA library was cloned in pGADT7 (carrying a Trp1 selection gene) and transformed into AH109 yeast strain (containing His3, Ade2, lacZ and Mel1 selections; Clontech, BD Biosciences, Pao Alto, CA). Cpne4 vWA domain was cloned into pGBKT7 (carrying Leu2 selection gene) and transformed into competent Y187 yeast strain (lacZ and Mel1 selection). The yeast mating experiment was performed as per the Two-hybrid library screening protocol for yeast mating (Clontech). Briefly, one colony of Y187 transformed with pGBKT7+ Cpne4 vWAdomain was inoculated in 50 ml of Tryptophan (Trp) selection media and incubated at 30°C overnight. The following day, the culture media was centrifuged, and the pellet re-suspended in 5ml Trp selection media. 45ml of YPDA media (Yeast extract Peptone Dextrose media supplemented with adenine(Ade)) was added to it. 1ml of cDNA library (titer = 5 x 10^7^ cfu/ml) was thawed in a water bath at room temperature and added to the above and allowed to mate for approximately 24 hours at 30°C with slow shaking at 50 rpm. The next day, the presence of mated, diploid yeast cells was checked under a light microscope. The culture was then centrifuged, and the pellet resuspended in 15 ml 0.5X YPDA. 300ul aliquots of the entire 15 ml mating culture was spread on 15 cm quadruple selection agar plates (with selection for Trp, leucine (Leu), Ade and histidine (His) and X-alpha-Gal reporter) and grown for about 5 days at 30°C. Small scale positive and negative control matings were also performed. For positive control, pGBKT7-53 encoding the p53 protein and pGADT7-T encoding the SV40 large T antigen protein were transformed into Y187 and AH109, respectively. For negative control, empty pGBKT7 and pGADT7, with no gene insertions, were transformed into Y187 and AH109, respectively. For the positive control mating, one colony each from Y187 + pGBKT7-53 and AH109 + pGADT7-T were inoculated in 500ul 2X YPDA and incubated overnight at 30 degrees at 200rpm. Similarly, for the negative control mating, Y187 + empty pGBKT7 and AH109 + pGADT7 were inoculated in 500ul 2X YPDA and incubated overnight at 30 degrees at 200rpm. The following day the control cultures were spread in 1:10, 1:100 and 1:1000 dilutions on separate single (Leu or Trp), double (Leu and Trp) or quadruple selection agar plates and grown for 3–5 days at 30°C.

After 4 days of selection, 241 pale blue colonies were picked for confirmation. They were streaked separately on fresh agar plates with quadruple selection. Six colonies grew into blue colonies after 3–4 days. For both positive and negative controls, the colonies appeared on the single selection and double selection plates. But on the quadruple selection plates, blue colonies appeared only on positive control and there were no colonies for negative control.

Colony polymerase chain reaction (PCR) was performed on the six selected colonies, and PCR products were extracted from the gel and Sanger sequenced (Eurofins, Luxembourg). Inserts were identified by BLAST (basic local alignment search tool)search against the NCBI mouse transcriptome database.

### 2.6 Co-immunoprecipitation

HEK293 cells were co-infected with HA-eGFP-Cpne4-vWAdomain construct (described in section 2.1) and Flag- tagged target protein or protein domain identified from Y2Hanalysis. 24 hours after the transfection, the cells were washed three times with 1X PBS. 300 ìl lysis buffer (50mM Tris-HCl, 150mM NaCl, 0.5% NP40 and 1mM ethylenediaminetetraacetic acid (EDTA)) containing protease inhibitor (Roche, Basel, Switzerland) and 0.2M phenylmethylsulfonyl fluoride was added to each well and incubated on ice for 15 minutes. The lysate was then collected in 1.5ml tubes and centrifuged at 14000g for 10 minutes to remove any debris. Meanwhile, magnetic beads were washed with 1X PBS and incubated with Flag antibody for 30 minutes, with end-to-end rotation at 4°C. The beads were again washed with 1X PBS to remove any unbound antibody and the supernatant from the cell lysate was added to it. These were incubated overnight with end-to-end rotation at 4°C. The next day, the beads were washed four times and PBS + Laemmli buffer (62.5mM Tris-HCl, 2% Sodium Dodecyl Sulfate (SDS), 10% glycerol, 5% beta-mercapto-ethanol, bromophenol blue) was added to the beads. The beads were boiled for 5 minutes at 70°C. The beads were separated on a magnetic rack and the supernatant loaded on a 10% SDS-Polyacrylamide gel electrophoresis (SDS-PAGE) gel. The gel was allowed to run until the dye front reached the bottom of the gel. The separated proteins were then transferred to 0.2 μm polyvinylidene difluoride (PVDF) membranes and processed further for Western blotting.

### 2.7 GST pulldown from retina

Glutathione S- transferase (GST) tagged Cpne4 protein was synthesized from bacteria as described before [31]. GST tagged Cpne4-vWAdomain (GST-Cpne4-vWA) and GST proteins were also synthesized similarly. 19 wild-type retinas (C57Bl6 and SV129) were homogenized using a glass homogenizer, in cold lysis buffer (RIPA buffer: 50mM Tris-HCl, 150mM NaCl, 1mM EDTA, Complete protease inhibitor (Millipore Sigma, Burlington, MA; Catalog no. 11697498001)). NP40 was then added to the lysate to a 0.5% final concentration. Glutathione- tagged magnetic beads were added to the lysate and incubated at 4 degrees with end-to-end rotation for 2 hours. The magnetic beads were removed, and the lysate centrifuged at 700 g for 10 minutes to remove any debris. The lysate was then kept on ice until further process. Equimolar amounts of GST, GST-vWA domain and GST-Cpne4 were incubated with glutathione tagged magnetic beads and incubated with end-to-end rotation at 4°C for 2 hours. The supernatant was discarded, and beads washed three times with PBS. Equal volume of cleared retina lysate (600 ìl each) was added to each of the above three tubes and incubated overnight at 4°C with end-to-end rotation. The following day, the supernatant was discarded, and the beads were washed four times with PBS. 30 ul of glutathione elution buffer was added to each of the tubes and incubated at room temperature for 10 minutes with end-to-end rotation. Laemmli buffer was added, and the samples boiled at 70°C for 5 minutes. The beads were separated, and the samples loaded onto 4–15% gradient SDS-PAGE gels. The samples were allowed to run on the gel until the dye front reached the bottom and proteins were visualized using Coomassie blue staining. Full lanes were cut out of the gel for each of the three samples, cut into smaller band size pieces and collected in separate tubes. Three such replicates were prepared each for GST, GST-Cpne4-vWAdomain and GST-Cpne4 pull-downs. The cut bands were then processed further for liquid chromatography mass spectrometry (LC-MS). An additional three replicates also prepared similarly, transferred to PVDF membranes directly after SDS-PAGE and checked with specific antibodies by western blotting. See the section on western blotting for further details.

### 2.8 Mass spectrometry

For sample preparation for LC-MS, Coomassie blue stained protein gel bands were first de-stained with 30% ethanol solution until the gel pieces became transparent. The gel pieces were then de-stained in 65% methanol + 10% acetic acid solution for 30 minutes. The destaining solution was removed and bands were washed with 100 mM triethyl ammonium bicarbonate (TEAB) solution for 5 minutes. 250 ul dehydration solution (75% acetonitrile in 100 mM TEAB) was added and the bands were agitated for 10 minutes at room temperature. The dehydration solution was removed, and the bands were air-dried for a couple of minutes. The gel pieces were then dehydrated with reducing solution (10 mM Tris(2-carboxyethyl)phosphinein 100 mM TEAB) for 45 minutes at 56°C. This solution was replaced by dehydration solution and bands incubated for 10 minutes at room temperature. The bands were then air dried and alkylation solution (20 mM iodoacetamide in 100 mM TEAB) was added and incubated for 30 minutes at room temperature. The gel bands were washed again and dehydrated again in the dehydration solution until the gel bands shrunk to half the size. The gel bands were air dried briefly and a trypsin solution was added to the bands, enough to cover the bands. The bands were left on ice for 5 minutes and more trypsin was added as needed. After 5 minutes, the trypsin solution was removed and 100 mM TEAB was added to tubes, enough to cover the bands. The tubes were incubated at 37°C for overnight digestion. The next day, the trypsin solution was removed, and digested peptides transferred to new tubes separately for each sample. 150ul of extraction solution (75% acetonitrile, 0.1% formic acid) was then added to each of the tubes and agitated for 10 minutes at room temperature. A short spin was done at 10,000 g and the gel pieces were saved. The extracts were then vacuum dried and 25 ul 1% trifluoroacetic acid was added to each tube. This was followed by a peptide clean-up as per the zip-tip protocol (Millipore Sigma; Catalog number C18 ZTC18S008). 20ul of 0.1% formic acid (in acetonitrile) was aspirated in the zip-tip and discarded. 20ul of 0.1% formic acid (in water) was similarly aspirated and discarded. The peptide extract was then aspirated 7–10 times, to let the peptides bind to the zip-tip column. This was followed by washing the zip-tip three times by aspiring 20 ìl of wash solution (0.1% formic acid in water) and dispensing it. To elute the bound peptides, 20 ìl of elution buffer (75% acetonitrile + 0.1% formic acid) was aspirated and dispensed in a tube. This step was repeated five times and each time eluate was collected in the same tube. The tubes were then dried in a speed vacuum. 20ul of 2% acetonitrile + 0.1% formic acid solution was added to the dried peptide digest and the tubes were vortexed and centrifuged. The solution was then transferred to LC vials for LC-MS analysis.

Desalted tryptic peptides were analyzed using nanoscale liquid chromatography tandem mass spectrometry (nLC- MS) and Ultimate 3000-nLC online coupled with an Orbitrap Lumos Tribrid mass spectrometer (Thermo Fisher Scientific, Waltham, MA). Peptides were separated on an EASY-Spray Column (Thermo Fisher Scientific; 75 mm by 50 cm inner diameter, 2-mm particle size, and 100-Å pore size). Separation was achieved by 4 to 35% linear gradient of acetonitrile + 0.1% formic acid for 90 minutes. An electrospray volt- age of 1.9 kV was applied to the eluent via the EASY-Spray column electrode. The Orbitrap Lumos was operated in positive ion data- dependent mode. Full-scan MS was performed in the Orbitrap with a normal precursor mass range of 380 to 1500 m*/z (*mass/charge ratio) at a resolution of 120,000. The automatic gain control (AGC) target and maximum accumulation time settings were set to 4 × 105and 50 ms, respectively. MS was triggered by selecting the most intense precursor ions above an intensity threshold of 5 × 10^3^for collision-induced dissociation (CID)–MS fragmentation with an AGC target and maximum accumulation time settings of 5 × 10^3^ and 300 ms, respectively. Mass filtering was performed by the quadrupole with 1.6 m/z transmission window, followed by CID fragmentation in the ion trap (rapid mode) and collision energy of 35%. To improve the spectral acquisition rate, parallelizable time was activated. The number of MS spectra acquired between full scans was restricted to a duty cycle of 3seconds.

Raw data files were processed with the Proteome Discoverer software (v2.4, Thermo Fisher Scientific), using Sequest HT (Thermo Fisher Scientific) search node for carbamylated peptide/protein identifications. The following search parameters were set: protein database UniProtKB/Swiss-Prot*Mus musculus* (17,033 sequences release 2020_10) concatenated with reversed copies of all sequences; MS1 tolerance of 12 ppm: ion trap detected MS/MS mass tolerance of 0.5Da; enzyme specificity set as trypsin with maximum two missed cleavages; minimum peptide length of 6 amino acids; fixed modification of Cys residues (carbamidomethylation); variable modification of methionine oxidation and acetyl on N terminus of protein. Percolator algorithm (v.3.02.1, University of Washington) was used to calculate the false discovery rate (FDR) of peptide spectrum matches (PSM), set to a q-value <0.05)

In order to identify retinal proteins that bind differentially to full-length Cpne4 or Cpne4-vWA domain, we compared the peptides pulled down by either GST-Cpne4-vWA, GST-Cpne4 or GST alone (as a control) and identified in the previous step. Of the 2119 proteins pulled down in either of the three conditions, we selected for further analysis those that were represented in all three replicates of at least one condition. Differential display analysis was performed using the R package “DEP” (DEP 1.12.0, 10.18129/B9.bioc.DEP; [34]). Missing values were replaced with 0, and data was normalized using VSN normalization. Pairwise comparisons for GST vs. GST-Cpne4 and GST-Cpne4-vWA samples were performed at thresholds of 0.05 FDR and 2-fold change.

### 2.9 Western blotting

Western blotting (WB) was done as described before [23]. Briefly, the PVDF membranes were washed with Tris buffered saline with 0.1% Tween20 (TBST). The membranes were then incubated in 5% milk (in TBST) for 1 hour at room temperature. Primary antibody solution prepared in 5% milk was added to the respective membranes and incubated overnight at 4C on a rocker shaker. The next day, the membranes were washed three times in TBST and secondary antibody solution (in 5% milk) was added. The membranes were then incubated at room temperature for 1 hour, followed by three washes with TBST. The membranes were exposed to Super signal chemiluminescence (Thermo Fisher Scientific) for 5 minutes and images taken on a gel dock (Bio-Rad, Hercules, CA). The details of primary antibodies used are given in Table 1.

### 2.10 Statistical analysis

The statistical analysis for comparing the aspect ratios of Cpne4 transfected HEK293 cells with C2 domains or vWA domain transfected HEK293 cells and varicosity areas in Cpne4 with control infected RGCs were done using Kolmogorov-Smirnov 2 (KS2) test, using MATLAB. For measuring colocalization of Cpne4 to interacting proteins in co-transfected HEK293 cells, Spearmann’s correlation coefficient (R) was measured using the Coloc2 tool on ImageJ.

Numbers of cells or regions of interests (ROIs) measured and p-values are given in the corresponding Results sections.

## 3. Results

### 3.1 Cpne4 transfection induces morphology changes *in-vitro*

To evaluate the subcellular distribution of Copine4 or its domains, we transfected expression vectors containing full length Cpne4, C2 domains or vWA domain tagged with HA (Fig 1A and 1B), in conjunction with a plasma membrane attached eGFP variant (meGFP) into HEK293 cells. Full length Copine4, C2 domains or vWA domain were detected in the nuclei, cell body as well as plasma membrane of the cells (Fig 1C) as indicated by both HA and Cpne4 staining. As previously reported, full length Cpne4 induces morphological changes (Fig 1C top panel; n = 2 experiments)- formation of elongated processes [31]. C2 domain or vWA domain constructs also induce formation of processes occasionally, although morphological changes were not as prominent and widespread as with Cpne4 transfection (Fig 1C middle and bottom panel; n = 2 experiments each; S1 Fig).

A morphometric analysis to determine the aspect ratio of Cpne4, C2 domains or vWA domains transfected HEK293 cells was done as described before [31]. This analysis shows a significantly higher aspect ratio for Cpne4transfected cells as compared to either C2 domains or vWA domain transfected cells (Fig 1D). The mean and median values and statistical analysis are given in Table 2.

**Table 2:** Aspect ratios of transfected HEK293 cells.

Plasmid transfected	Mean Aspect ratio	Median Aspect ratio	t-test p value (vs. Cpne4)	KS2 test p value (vs. Cpne4)
Cpne4 (n = 14 cells)	2.57	2.62		
VWA domain (n = 17 cells)	1.86	1.15	7.85e-10	1.86e-08
C2 domains (n = 16 cells)	1.3	1.24	7.40e-07	7.06e-06

### 3.2 Cpne4 infection induces morphology changes *in-vivo* in RGCs

**Fig 2:**
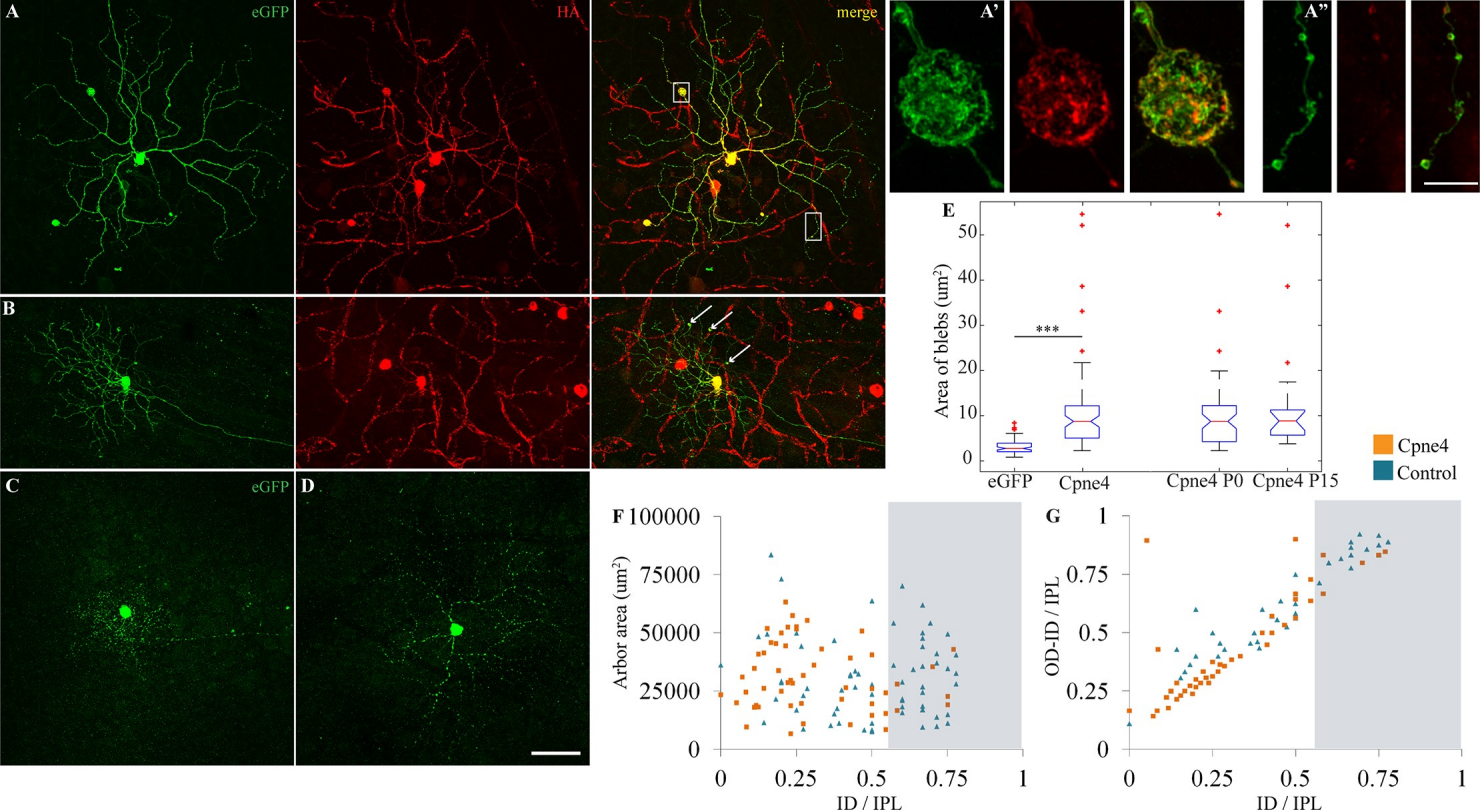
Cpne4 virus infections lead to ‘bleb’ formation on dendrites of Brn3b+ RGCs. (A, B) Intraocular injections of AAV1-Cpne4 viruses were done in P0 or P14 Brn3b^Cre/WT^ mice eyes. Blebs (indicated by *boxes* and *arrows*) were seen occasionally on dendrites of Cpne4 infected RGCs. For controls, AAV1-eGFP was similarly injected in P0 Brn3b^Cre/WT^ mice (C, D). No blebs were observed in the control infected RGCs. Airy scan images for one of the blebs (A’) and adjoining dendrites (A”). (E) Measurement of the ‘bleb’ areas for Cpne4 infected RGCs as compared to regular varicosities on controls shows a significant difference between them while no difference between P0 and P15 Cpne4 infected retinas. (F, G) Areas and lamination measurements indicate no differences between the Cpne4 transfected RGCs as compared to control transfected RGCs. (ID and OD: Inner and outer distance- distance between innermost or outermost tip of RGC dendrites and the INL; IPL: Width of the IPL). Scale bar for A-C: 50μm; Scale bar for A’, A”: 5μm.***, p< 0.001.

Given the morphological defects observed when overexpressing Cpne4 in HEK293 cells in culture, we asked whether overexpressing Cpne4 in RGCs will affect RGC morphology. We therefore infected either P0 or P15 Brn3b^Cre/WT^ mice with AAV1 vectors co-expressing HA-tagged Cpne4 and meGFP in a Cre-dependent manner. Membranes from infected Brn3b^Cre/WT^ RGCs were labelled with eGFP (green), revealing axonal arbors, cell bodies and dendrites (Fig 2A and 2B). In contrast, Cpne4 expression as seen by HA (red) labeling was largely confined to the cell body and dendrites, and only rarely reached into the axon (Fig 2A and 2B; n = 6 animals, 72 cells). Interestingly, several large varicosities were observed on dendrites of Cpne4 infected Brn3b^Cre/WT^ RGCs. These large varicosities (we call these ‘blebs’, Fig 2A and 2A’) can be easily distinguished from regular varicosities, as seen on the RGC dendrites in Cpne4 infected cells (Fig 2A and 2A”, S2A Fig, n = 6 animals, 72 cells), or on the dendrites of control RGCs infected solely with AAV1-eGFP (Fig 2C and 2E; area = 10.7± 0.61μm^2^ for Cpne4 versus 3.12± 0.18μm^2^ for controls, KS2 test p value < 0.01; controls n = 8 animals, 73 cells). The size and number of blebs varied significantly across the infected RGCs, and bleb size tended to increase with RGC dendritic arbor area (S2B and S2C Fig). A comparison between the P0 and P15 Cpne4 infected RGCs suggest no differences in the bleb areas (P0 mean area = 9.95± 1.11μm^2^, P15 mean bleb area = 15± 3.75μm^2^; p = 0.2), suggesting that the process disrupted by Cpne4 overexpression is still active in nearly adult animals (P15), as opposed to the early stages of dendrite formation (P0). A high-resolution airy scan imaging revealed that blebs contained large amounts of Cpne4, as well as cell membranes, as revealed by membrane attached eGFP. Besides the formation of large varicosities or blebs, Cpne4 over-expression into RGCs did not cause any changes in the arbor area or stratification when compared to controls infected with eGFP only (Fig 2F and 2G).

### 3.3 Yeast two hybrid analysis reveals Cpne4 vWA domain interaction with Morn2, HCFC1 and Tox3 domains

**Fig 3:**
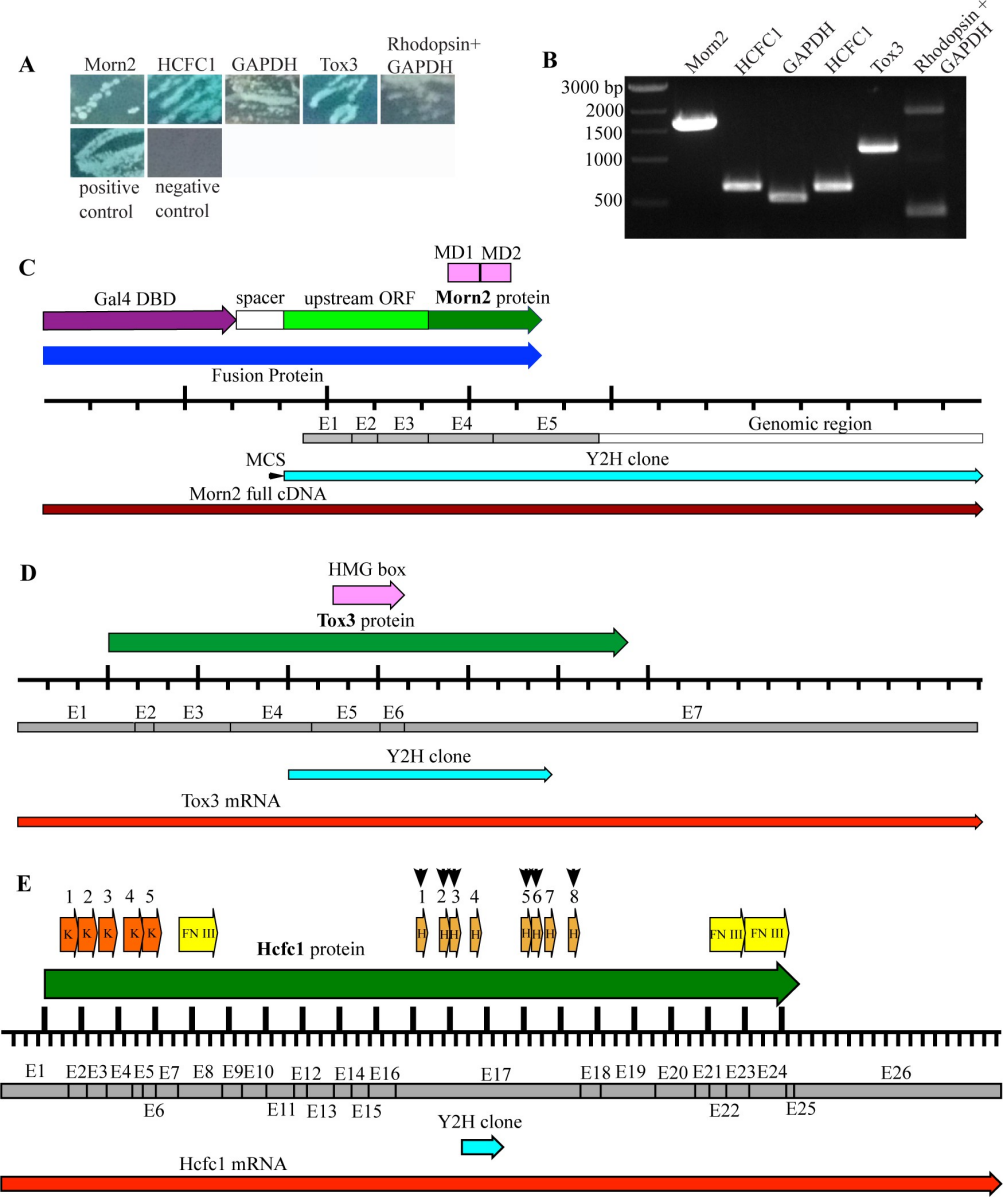
Yeast 2 hybrid analysis shows 5 proteins interacting with Cpne4 vWAdomain. (A)A Gal4 based yeast two hybrid analysis on the adult mouse retina cDNA library with Cpne4 vWA domain as bait protein showed five positive interactions (blue colonies). (B) Gel image of colony PCR performed on the positive clones from the yeast two hybrid shows the corresponding interacting DNA sequences. The identity of these DNA sequences as confirmed by the Sanger sequencing followed by BLAST analysis are shown as labels on the lanes. (C-E) Sequence maps of the interacting peptides as identified by Sanger sequencing followed by BLAST analysis are shown for- Morn2 (C), Tox3 (D) and HCFC1 (E). The relative positions of the actual interacting region (*light blue*) are indicated on the respective protein (*dark green*) and the corresponding mRNA (*red*) or prey Gal4 DBD- cDNA (*maroon*) sequences, and the exons (*gray*) are also indicated on the respective maps.The black ruler with tick marks indicates 100 amino acids (upward ticks) or 100 base pairs (downward ticks).The various domains and regions are as labeled on the maps. E: Exon; MD: Morn domain; MCS: Multi cloning site; K: Kelch domain; FN III: Fibronectin type III domain; H: Cleavage site, Gal4 DBD: Gal4 DNA binding domain derived from prey library.

The domain structure of Copines, and previous work on other family members suggests that C2 domains facilitate Ca^2+^ mediated membrane attachment, while vWA domain mediates interactions with other proteins. We therefore used a Gal4 based Y2H system using the Cpne4 vWA domain as bait protein and an adult mouse retina cDNA library as prey, in order to identify potential retina-specific interactors of Cpne4. Only five clones selected from the initial screening confirmed upon replating (Fig 3A). A colony PCR (Fig 3B) and subsequent Sanger sequencing on the PCR products identified the clones as domains of five proteins- 2 clones of HCFC1, 1 each of Morn2, Tox3 and GAPDH and a mixed clone consisting of Rhodopsin and GAPDH (Fig 3B). Since Rhodopsin is not expressed in the inner retina and RGCs, where Cpne4 is expressed, and GAPDH is a housekeeping gene, these two interactions were not pursued for further analysis.

The Morn2 Y2H clone contained 1474 base pairs (Fig 3C). It contains an ORF in frame with the Gal4 DNA binding domain (Gal4 DBD) that encodes 180 amino acids. BLAST search against NCBI database identified the Morn2 NM_194269 transcript as the top match, consisting of 662 base pairs and including 5 exons. The homology of the Y2H clone spans base pairs 41–664 on the Y2H clone which correspond to the base pairs 24–647 on NM_194269 mRNA transcript. However, the Morn2 Y2H clone extends for another 810 base pairs beyond the end of exon5 into the genomic DNA (Morn2 mouse gene in NCBI database, reference number NC000083). Morn2 has several other alternative splicing variants extending past the exon 5 in NM_194269 that include three alternative exons 6 at various distances from exon 5. Our Y2H clone would include the intron 5 and exon 6 as annotated in variant XM_036160654 in the NCBI database and would represent a novel alternative splicing variant.

The canonic Morn2 Protein comprises of 79 amino acids containing two MORN (Membrane Occupation and Recognition Nexus) motifs [35–37], encoded by exons 4 and 5 in NM_194269 transcript. Thus, the alternative splice variants on the 5’ and 3’ end do not affect the predicted amino acid composition.

The Y2H prey vector encodes the Gal4 DBD attached to the prey library via a 34 amino acid spacer containing a H2A tag and the multicloning site. The Morn2 Y2H clone contains 304 bp upstream of the Morn2 ATG (transcription start site), encoding an additional 101 amino acids, ie, an upstream open reading frame (ORF) which is in frame with the Gal4 DBD, via the spacer. Thus, the full fusion protein comprises, in order from N terminus- the Gal4 DBD, spacer (34 amino acids), upstream ORF (101 amino acids) and the previously reported Morn2 protein (79 aa). It should be noted that in the Morn2 mRNA (NM_194269) the same upstream ORF is open but misses a methionine initiator codon. However, this potential upstream ORF bears strong amino acid homology with Morn2 orthologues in other species, including flatworms (*Dugesia japonica*) [36], and thus, may be functionally relevant.

BLAST alignment of our 938 base pairs Y2H Tox3 clone (Fig 3D) against NCBI database yielded NM_172913, a Tox3 transcript, consisting of 7 exons as the top hit. Base pairs 40–916 of the Y2H Tox3 clone are a 100% identical to bases 903–1778 of NM_172913, covering the end of exon 4 through the beginning of exon7. They encode for amino acids 200–492, that span the HMG box of Tox3 protein. HMG box of Tox3 is required for Ca2+ dependent regulation of transcription through its interaction with phosphorylated CREB [38].

The HCFC1 clone (Fig 3E) contained a 434 base pair sequence. Of this, base pairs 40–375 correspond to base pairs 3750–4085 of NM_008224.4 HCFC1 transcript. The remaining 59 base pairs contain a polylinker region. The NM_008224 transcript has 26 exons, for a total protein length of 2045 amino acids. The Y2H clone is completely included in exon 17, and overlaps with HCF repeat 4, thus lying in between the clusters of self-cleavage motifs. HCF repeat 4 in HCFC1 is one of the six 26 amino acids repeats which get cleaved in the cytoplasm, following which the N and C terminal peptide fragments of HCFC1 attach non-covalently, are transported to the nucleus and participate in cell cycle, cell growth, cytokinesis and other functions [39].

Complete sequences of the regions of Morn2, HCFC1 and Tox3 interacting clones are given in S1 Text.

### 3.4 Cpne4 vWA domain interacts with HCFC1, Morn2 and Mycbp2 in HEK293 cells

**Fig 4:**
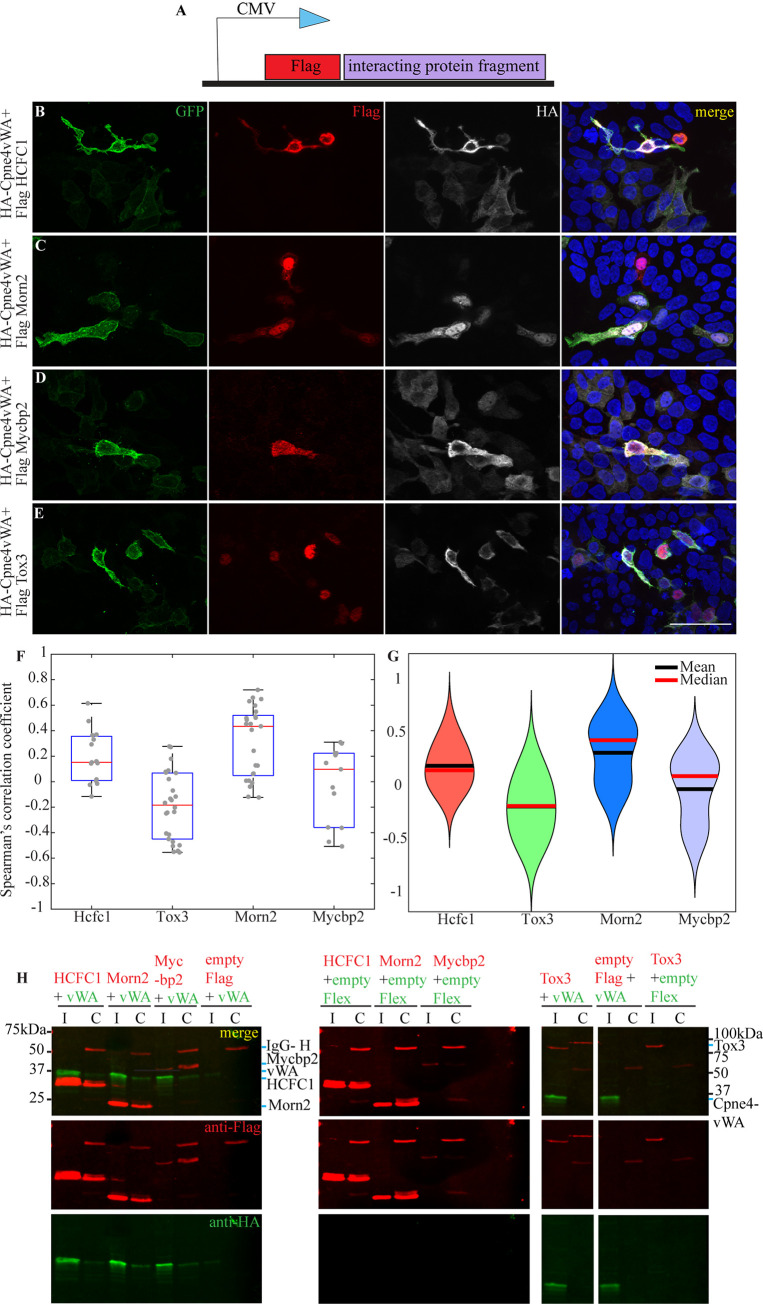
Cpne4 vWAdomain was pulled down with HCFC1, Morn2 and Mycbp2, but not by Tox3. (A) Map of a Flag clone consisting of a Flag tag followed by the interacting regions of HCFC1, Morn2, Mycbp2 and Tox3. (B-E) Representative images of HEK293 cells co-infected with the expression construct for Cpne4-vWAdomain (HA-Cpne4vWA) and Flag- tagged interacting protein. (F, G) Box plots and violin plots indicate the Spearmann’s correlation coefficient for co-localization between Morn2, HCFC1, Tox3 or Mycbp2 with Cpne4. Data points are shown as gray circles, medians are indicated by red lines and means by black lines. (H) Western blot images of pull down from co-transfected HEK293 cells using Flag antibody show the total lysate (*I*) and the co-immunoprecipitated (*C*) proteins. Cpne4-vWA (*green*; 31kDa) was pulled down with Flag- HCFC1 (23.5kDa), Morn2 (21kDa), Mycbp2 (42kDa) but not Tox3 (66kDa; all appear as *red* bands).Scale bar: 50μm.

We next sought to validate our candidate Cpne4 interactors (HCFC1, Morn2 and Tox3 domains) by co-immunoprecipitation. In addition, we surveyed candidate Cpne4-vWA interactors identified in a previous Y2H analysis using a mouse embryonic cDNA library [32]. By exploring our RNA sequencing analysis [10], we found that some of these proteins (Bicd2, Pitpnm2, Sptbn1 and Mycbp2) were indeed expressed in RGCs (S3 Fig). We cloned the interacting peptide regions from our candidates (HCFC1, Morn2 and Tox3) as well as the matches from a previous study [32] (Bicd2, Pitpnm2, Sptbn1 and Mycbp2) downstream of the Flag tag in an eukaryotic expression vector (Fig 4A) and co-transfected them with the HA-tagged Cpne4-vWA in HEK293 cells. For simplicity, we refer to the interacting protein domains with the names of their source proteins in rest of the text, as well as in the corresponding figures.

The colocalization of these proteins was observed by immunostaining (Fig 4B–4E; n = 6, 5, 4 and 4 for Morn2, HCFC1, Mycbp2 and Tox3 respectively). While HCFC1, Mycbp2 and Morn2 were localized in the cell body of the cells, Tox3 was always localized in the nuclei (Fig 4B–4E).

Colocalization analysis between Cpne4 and potential interactors showed that both HCFC1 and Morn2 had a positive correlation with Cpne4 (mean Spearmann’s correlation coefficient R = 0.19 for HCFC1, n = 15 ROIs; mean R = 0.32 for Morn2, n = 25 ROIs) whereas Tox3 did not (mean R = -0.19, n = 26 ROIs; Fig 4F and 4G). Furthermore, a pull-down analysis with Flag antibody showed that the identified Morn2 and HCFC1 domain can interact with the Cpne4 vWA domain while Tox3 does not (Fig 4H; n = 5 for HCFC1, n = 4 for Morn2, n = 3 for Tox3). Of the four candidates selected from a previous study [32], only Mycbp2 exhibited a small degree of colocalization with Cpne4 (mean R = 0.07, n = 13 images; Fig 4F and 4G). Mycbp2 also pulled down the Cpne4 vWA domain (Fig 4H, n = 4). The other candidates—Bicd2, Pitpnm2 and Sptbn1—did not pull down the Cpne4 vWA domain (S4 Fig).

**Fig 5:**
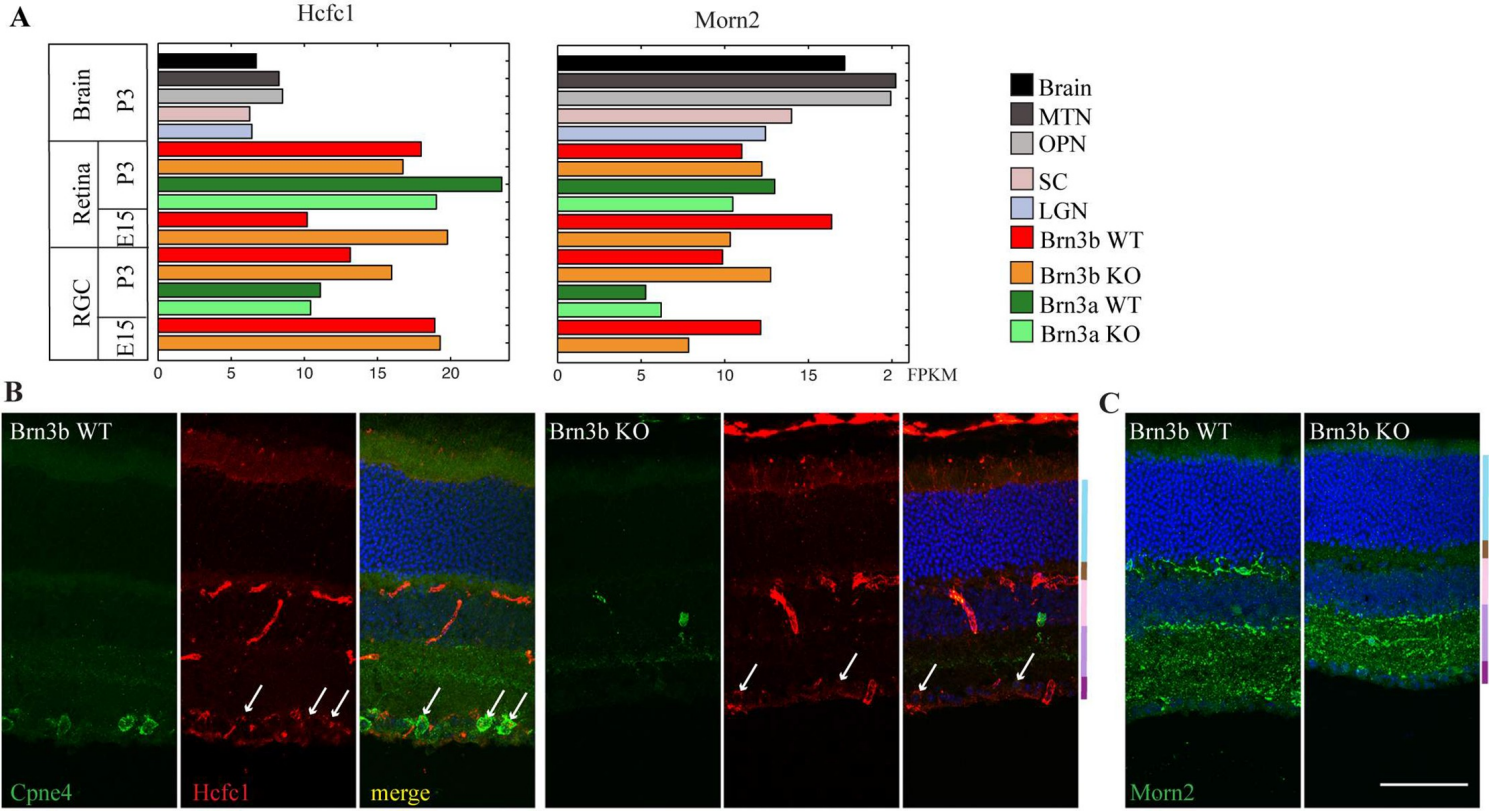
Expression of Morn2 and HCFC1 in retina. (A) RNA sequencing data shows FPKM (fragments per kilobase of transcripts per million mapped reads) for Morn2 and HCFC1 in visual brain areas (MTN:medial temporal nucleus, OPN:olivary pretectal nucleus, SC: Superior coliculus, LGN: Lateral geniculate nucleus), rest of the brain (Brain) Brn3a wild-type (WT) and knockout (KO) RGCs, Brn3b WT and KO RGCs and rest of the retina (Retina). Brain samples were obtained from P3 WT (wildtype) mice and retina samples from P3 or E15 mice.The genotypes for retina RGC samples are: Brn3b WT = Pax6α:Cre; *Brn3b*^*CKOAP/WT*^,Brn3b KO = Pax6α:Cre; *Brn3b*^*CKOAP/KO*^, Brn3a WT = Pax6α:Cre; *Brn3a*^*CKOAP/WT*^, Brn3aKO = Pax6α:Cre; *Brn3a*^*CKOAP/KO*^. Values for brain areas represent medians for three samples (LGN, SC), two samples (whole brain) or individual samples pooled from three animals (MTN and PTA). Retina values represent samples obtained from two pooled retinas, while RGC values are medians of two biological replicates each obtained from 6–8 retinas. Data is extracted from Sajgo et al., 2017(10). (B) HCFC1 (*red*) immunostaining in retina indicates some expression in RGCs in both the WT and Brn3b KO retinas. There is some co-labeling with Cpne4 (*green*) in the RGC cell bodies (*arrows*). (C) Morn2 (*green*) immunolabeling in the retina indicated mostly dendritic labeling in the IPL in both Brn3b WT and KO retinas. There is also some Morn2 labeling in the OPL of WT that is reduced in the Brn3b KO.Retinal layer color codes in B,C: ONL- *light blue*, OPL- *brown*, INL- *pink*, IPL- *purple*, GCL- *magenta*. Scale bar: 50μm.

RNA expression as observed by RNA sequencing analysis (Fig 5A) [1] reveals that HCFC1 and Morn2 are expressed in RGCs as well as the rest of the retina at E15 and at P3 (Fig 5A). Immunostaining for HCFC1 in the retina showed that it was localized in the Brn3b^+^ RGCs in the ganglion cell layer in both Brn3b WT as well as KO retinas (Fig 5B; n = 2). Immunostaining for Morn2 showed expression in the inner plexiform layer (IPL) and ganglion cell layer (GCL) in both Brn3b WT and KO retinas (Fig 5C; n = 2).

### 3.5 Mass spectrometry analysis of retinal proteins identifies potential Cpne4 interactions

**Fig 6:**
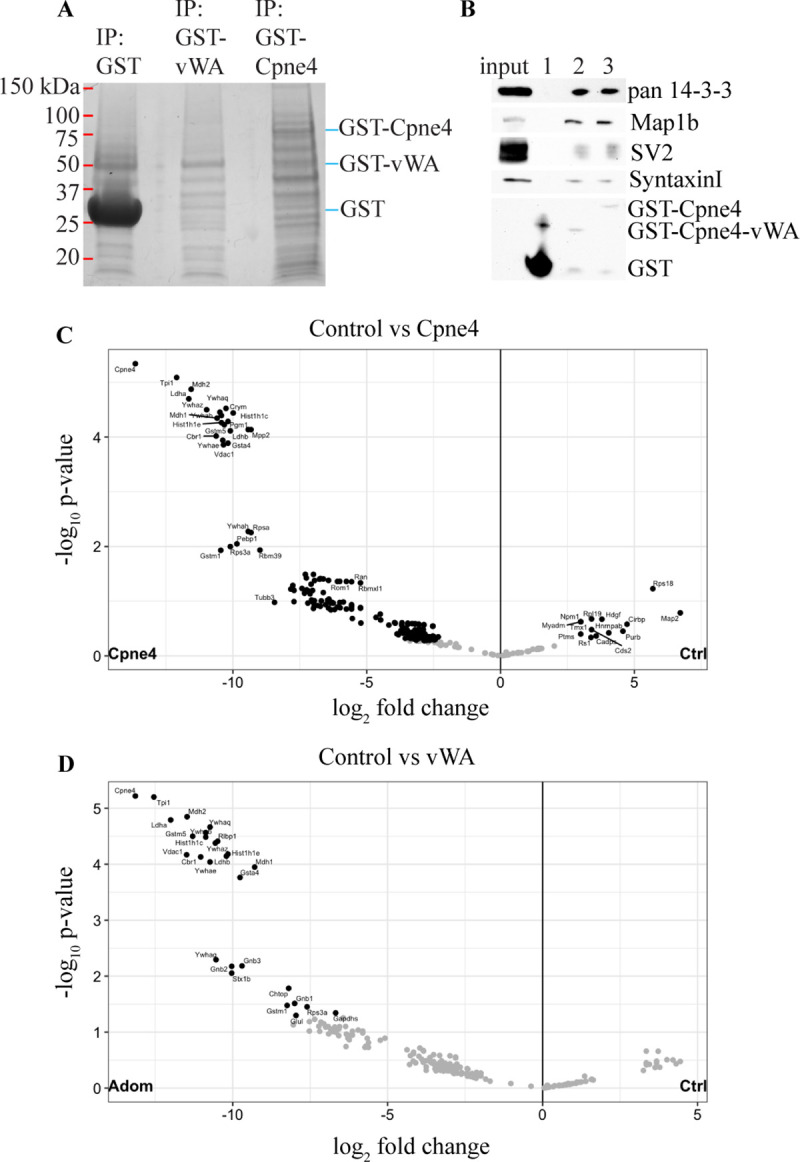
Pulldown with GST-Cpne4 or GST-Cpne4-vWA domain and LC-MS. (A) Representative SDS-PAGE gel for retina pull downs using GST- Cpne4, GST- Cpne4- vWA and GST only. (B) Representative Western blot image of some of the interactors from the pull-down experiment- pan 14-3-3 (30kDa); Map1b (325kDa); SV2 (95kDa) and Syntaxin1 (33kDa) as compared to GST (28kDa) controlfor retina pull downs using GST (*1*), GST- Cpne4- vWA (*2*) and GST- Cpne4 (*3*). (C) Volcano plot for proteins that had a significant interaction with GST- Cpne4 as compared to GST control. (D) Volcano plot for proteins that had a significant interaction with GST- Cpne4- vWA as compared to GST control.

Cpne4 protein interactions with the vWA domain may be modulated by the C2 domains. In order to identify potential native, full length Copine4 protein binding partners in the retina, we performed pulldown assays of mouse retina protein extracts with GST-vWA or GST-Cpne4 fusion proteins, alongside GST-only controls using Glutathione-Sepharose beads (Fig 6A). Each experimental condition was repeated in triplicate (19 retinas per replicate), and Glutathione eluates were loaded onto polyacrylamide gels, separated by electrophoresis, gel bands cut out and processed for LC-MS. A total of 2119 proteins were represented in the LC-MS results by at least one peptide (S1 Table). In order to increase the stringency of our screen, we considered for analysis only proteins that were represented by at least one peptide in all three replicates of at least one of the pull-down conditions (GST-Cpne4, GST-vWA or GST only; 278 total). Differential expression was performed using the Bioconductor R package DEP (see section 2.8), and a fold change of 2 and FDR of 5% were used as thresholds for differential expression. Based on these criteria, 27 proteins were enriched in both GST-Cpne4 and GST-vWA relative to GST controls, while 180 proteins were significantly enriched in GST-Cpne4 but not GST-vWA samples relative to GST controls (Fig 6C and 6D, S1 Table).

Gene ontology (GO) analysis performed on the 207 proteins that interacted with Cpne4 or Cpne4-vWA (S2 Table) identified 412 biological processes and 146 molecular functions significantly enriched (FDR p<0.05). Amongst the top 30 most significantly enriched biological processes (FDR < = 5.21 e-09) all but two were related to metabolic processes, including glycolysis, ATP or nucleotide metabolism. The two biological processes unrelated to metabolism but interesting from the perspective of Cpne4 involvement in cell morphology and differentiation were “regulation of localization” (FDR < = 2.32 e-11) and “regulation of transport” (FDR < = 4.29 e-10). Molecular functions were mostly associated with nucleotide binding, protein binding, enzymatic or catalytic activity. Of the 180 GO cellular components that appeared enriched amongst Cpne4 interactors, at the very top were compartments associated with myelin sheath, cytoplasm, synapse, plasma membrane bounded cell projection, and neuron projection (FDRs < = 2.84 e-19). Interestingly, the highest fold enrichment and low FDR for Cpne4 and its interactors also suggested its localization in the late endosome lumen. A GO pathways analysis (PANTHER GO pathways) reveals that potential Cpne4 interactions participate overwhelmingly in a variety of neuronal seven transmembrane receptor pathways (Muscarinic, metabotropic glutamate receptor, Opioid, Serotonin and Dopamine, etc.) at FDRs < = 8.03 e-05. PI3 Kinase and cytoskeletal regulation by Rho GTPase pathways were also significantly enriched. The complete list of all these suggested functions and locations from GO analysis are listed in S2 Table.

**Fig 7:**
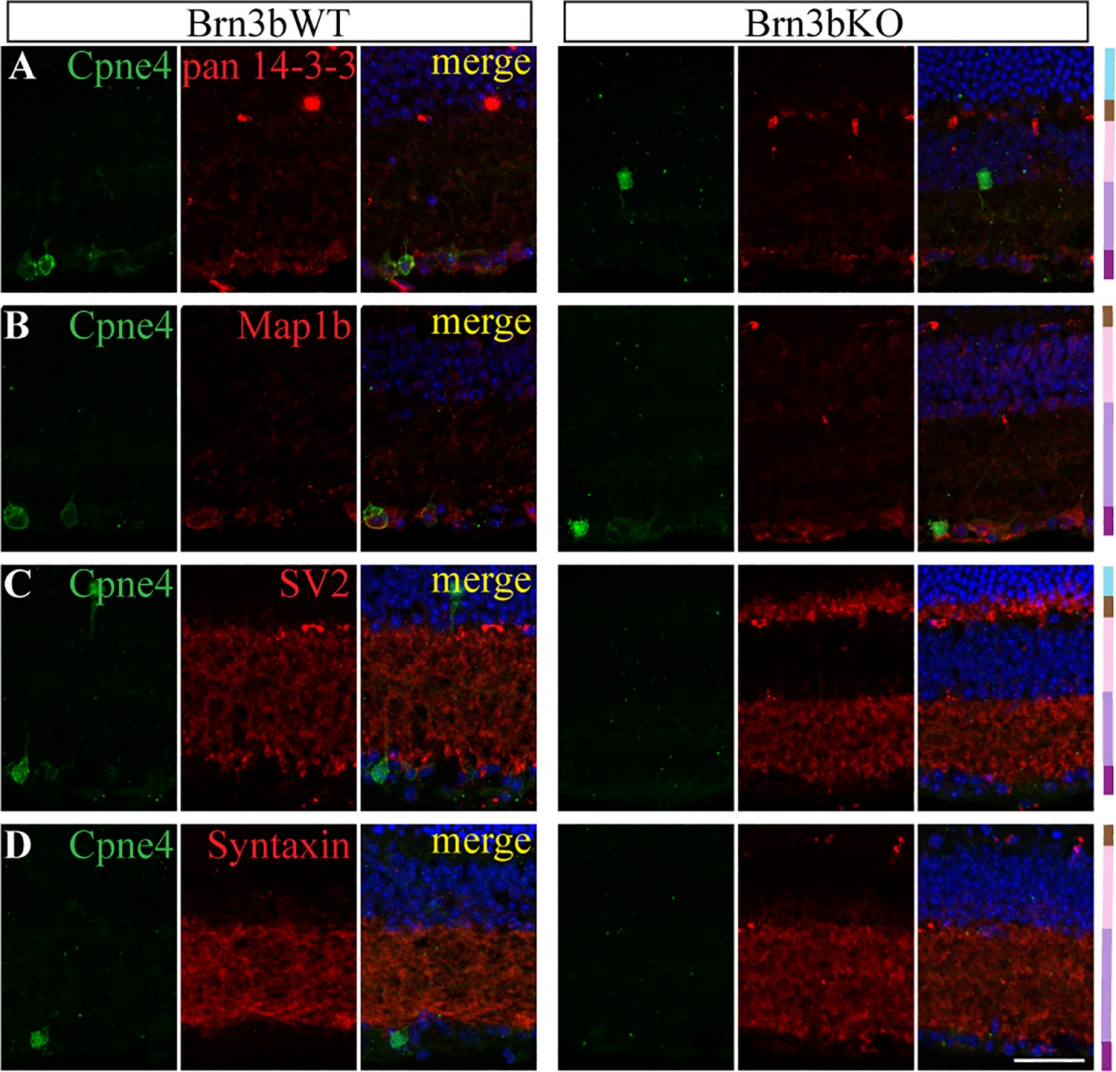
Immunostaining of LC-MS interactors with Cpne4 in retina. Representative images showing immunostaining of pan 14-3-3 (A); Map1b (B); SV2 (C) and Syntaxin (D; *red*) with Brn3b (*green*) in Brn3b WT (left panel) and KO (right panel). Retinal layer color codes: ONL- *light blue*, OPL- *brown*, INL- *pink*, IPL- *purple*, GCL- *magenta*. Scale bar: 50μm.

In order to validate our MS screen, we analyzed several proteins associated with some of the enriched processes: 14-3-3 family of proteins, Map1b, SV2 and Syntaxin-1 (S5 Fig). Their association with either GST-vWA or GST-Cpne4 in the retina was confirmed by Western blotting of pulldowns from retinal lysates (Fig 6B). RNA sequencing data showed that the RNA for these candidates was enriched in the retina (S3 Fig). Using immunohistochemistry, pan 14-3-3 and Map1b were found to colocalize with Cpne4^+^ in RGCs (Fig 7A and 7B; n = 3 retinas each) in Brn3b WT and KO retina. SV2 and Syntaxin were colocalized in the IPL of both Brn3b WT and KO (Fig 7C and 7D; n = 3 retinas each).

## 4. Discussion

Amongst Copine family members, Cpne4 has one of the highest mRNA expressions in the retina and is predominantly expressed in the RGC layer [31]. Cpne4 over-expression in RGCs lead to formation of large varicosities on dendrites but no other morphological changes to RGC dendritic arbor area or stratification. The morphological defects observed by overexpression in HEK293 cells require a full length Cpne4 protein, as neither C2 domains nor vWA domain can induce extensive process formation in isolation. Cpne4 may be involved in several distinct metabolic and signaling pathways, as revealed by our Y2H and retina proteome interaction studies. Several proteins can interact directly with Cpne4 including Morn2, HCFC1, 14-3-3 family, Map1b, Syntaxin1 and SV2.

### 4.1 Cpne4 regulates cytoskeletal structure in the cells

It is known that Brn3 transcription factors cause morphological changes in the retina during development and these changes are most likely achieved through their transcriptional targets [2,10,40]. Cpne4 is one such molecule that is regulated by both Brn3a and Brn3b [10]. Results from the current study and previously reported experiments show that over-expression of various Copines in vitro in HEK293 cells leads to elongation of the transfected cells and formation of processes [31]. Transfection with the dominant negative forms of Cpne4 however, did not result in substantial morphology changes. This suggests that the association of C2 domains and vWA domain are critical for inducing phenotypic changes in vitro. Interestingly, the GO analysis of Cpne4 and its interacting proteins suggests its localization in plasma membrane bounded cell projection, and neuron projection, among other locations indicating that it might be involved in cytoskeleton–plasma membrane interactions within the cells.

Large varicosities, similar to the ones observed on RGC dendrites following Cpne4 over-expression, are reminiscent of previously described dendrite swellings, under normal and pathogenetic conditions. In Drosophila larvae, dendrite arborizations of superficial sensory neurons are heavily remodeled during metamorphosis. Prior to pruning, the dendrites tagged for elimination experience marked calcium transients, followed by increased endocytosis and formation of varicosities, like beads on a string. The beaded dendrites are eventually resorbed prior to formation of the new arbor [41,42]. Similar large varicosities are also observed on the dendrites of neurons undergoing NMDA-induced excitotoxicity [43,44].

Other Copines have been previously shown to be involved in morphological changes. Cpne6 mediates cytoskeletal changes in the hippocampus during long term potentiation [26,27]. Cpne6 interacts with Rac1 to regulate the Rac1/LimK/Cofilin signaling pathway that further regulates microtubule polymerization and hence affects the spine formation in hippocampal neurons [26]. Cpne3 along with Rac1 is required for cell migration during tumor metastasis [45]. Copine A, a Copine homolog in Dictyostelium, interacts with actin filaments and regulates chemotaxis and adhesion in these organisms [24]. CPNA1, Copine homolog in C. elegans, is located at integrin adhesion sites in the muscle cells and acts as a linker between various myofilament proteins to maintain muscle stability [22].

Taken together, these studies suggest that Copines may mediate membrane–cytoskeletal interactions in a variety of contexts. It is not clear whether the large varicosities on RGCs indicate a role of Cpne4 in dendrite remodeling, or cytoskeletal changes during dendrite development of the retina. Expression of Cpne4 and several other Copine family members increases dramatically during the first two postnatal weeks, a period of intense RGC dendritic arbor growth, differentiation and maturation of dendritic arbor at the time of eye opening. However, the varicosities appeared irrespective of whether the Cpne4 was over-expressed at P0 when the eyes are closed, and the inner retina is still developing or at P14 when the eyes are open and pruning of the dendrites happens.

### 4.2 Cpne4 and endosomal/lysosomal/autophagosomal pathways

Y2H analysis on a retina cDNA library indicated Cpne4 interacts with Morn2. Morn2 RNA is expressed in the RGCs, and protein is expressed in the RGCs and their dendritic arbor in the IPL. Morn2 interacts with LC3 to recruit phagosomes against various bacteria in planarian flatworms [36,37]. Morn2 has also been seen to be important in adhesion of bacteria to cell membranes and eventually invasion of bacteria into the cells [46]. Other Morn repeat containing proteins with known functions are junctophilin, retinophilin (also known as Morn4 or Undertaker), PIPK (phosphatidylinositol monophosphate kinase), and Alsin2.

We and a previous study [32] have confirmed that Mycbp2 interacts with Cpne4. Amongst other functional connections (see below), Mycbp2 has a Ubiquitin ligase domain through which it can modulate autophagy and its involvement in axon guidance [47,48]. Rpm-1, the *C*. *elegans* homolog of Mycbp2, regulates AMPA receptor trafficking by regulating the degradation of DLK1 which further regulates Rab5 endosomal protein [49,50]. Rab5 endosomes are required for dendritic branching [51].

Copine A, in Dictyostelium also associates with vacuoles, endolysosomal organelles and phagosomes in Ca^2+^ dependent way [52]. Cpne6 translocates to clathrin coated vesicles when Ca^2+^ concentration increases in the cells [14].Cpne1 has previously been reported to be involved in endosomal and autophagocytosis pathways. Specifically, Cpne1 together with AnnexinA1 and AnnexinA5 and is involved in calcium dependent autophagosomal degradation [53,54]. Cpne1 is also involved in endoproteolysis of NFƘB [55].

Further studies need to be done to see if Cpne4 is involved in similar function in the retina. One of the top hits for probable sub-cellular location of Cpne4 based on the GO analysis of its interacting proteins is the late endosome lumen. Another possible function of Cpne4 as indicated by GO analysis suggests its role in regulation of transport in the neurons. In this context, the dendritic varicosities induced in RGCs could be the result of disrupted vesicle trafficking. We note that super-resolution imaging of varicosities reveals accumulation of membranes, besides Cpne4 itself.

### 4.3 Cpne4 and neuronal maturation and synapse formation

Copines were initially discovered in the cell bodies and dendrites of hippocampus neurons and dendrites [18,25] and have been shown to be involved in a variety of functions in the brain. Cpne6 has recently been shown to be involved in long term potentiation [26,27] and suppression of spontaneous neurotransmission [56]. Cpne1 is upregulated during neural tube closure in mouse embryo, it regulates neural stem cell functions during development, and it is also required for progenitor cell differentiation of neurons in the hippocampus [28,29,57]. The role of Copines in the development or physiology of retina is unknown. But the interactions of Cpne4 with various proteins indicate several possible functions in the retina.

Several protein interactions with Cpne4 were observed with LC-MS analysis. Some of these that were particularly interesting and were expressed in the retina specifically in the inner retina were the 14-3-3 family, Map1b, Syntaxin1 and SV2. 14-3-3 family of proteins is one of the most abundant proteins in the central nervous system. 14-3-3Ɛ, that was found to interact with Cpne4, is previously known to bind to doublecortin (Dcx) protein, prevents its degradation which further prevents neurite formation by preventing the microtubules from invading the lamellipodia [58]. 14-3-3Ɛ and 14-3-3ζ are known to regulate neurogenesis and differentiation of neuronal progenitor cells in the cortex. This is achieved by regulating the catenin/Rho GTPase/Limk1/cofilin signaling pathway to promote F-actin formation [59]. 14-3-3ɣ regulates Cpne1 to enhance differentiation of hippocampal progenitor cells and neurite formation in these cells [29,60]. Map1b plays an important role in axon guidance and axonal branching during the development of the nervous system [61,62]. Synaptic proteins Syntaxin1 and SV2, also interacting with Cpne4, are required in neurotransmitter release at the presynaptic terminal in the retina [63,64]. Interestingly, according to GO analysis of Cpne4 and its interacting proteins, one of the highest probabilities of localization of Cpne4 is at the synapse. This also correlates with the previous reports on localization and interaction of Cpne6 with various SNARE proteins on the presynaptic membrane as well as PSD in the postsynaptic membrane [26,56].

The Y2H analysis revealed HCFC1 as an interaction of Cpne4. HCFC1 RNA and protein is also expressed in retina and RGCs. HCFC1 was initially discovered as a transcriptional co-activator that along with Oct1 forms a complex with VP16 from Herpes simplex virus and helps utilize the transcriptional machinery of the host cell for replicating the virus [65]. In neurons, over-expression of HCFC1 leads to neuronal maturation and also limits neurite growth [66]. Mutations in HCFC1 protein are associated with Non syndromic Intellectual Disability in humans [67].

Mycbp2, and its orthologs in flies (*D*. *Melanogaster*), worms (*C*. *elegans*) and fish (*D*. *Rerio*) is a large protein containing several well characterized domains, through which it links axon guidance pathways (Netrin and Slit signaling), TGFβ and mTor signaling, to G-protein, Adenylate Cyclase, and MAPK pathways [47]. Loss of function mutations in flies (Highwire), worms (RPM-1) and mice (Magellan) result in severe axon guidance (abnormal sprouting) and synapse formation deficits (weak presynaptic elements) in both sensory and motor neurons. Highwire, the homolog of Mycbp2 in Drosophila is also required for synapse formation [68,69]. In retina, Mycbp2 RNA expression is observed in RGCs and other cell types and is required for axonal growth cone formation [70]. Knocking out Mycbp2 prevents RGC axons from reaching the thalamus and mis-targeted axons in mouse LGN [71–73].

Finally, we should point out that none of the targets identified in the Y2H screen were recovered from the GST-Cpne4-vWAdomain pull-down proteomics experiments. Nevertheless, co-immunoprecipitation experiments confirmed several targets identified through both approaches. Thus, while each approach may reveal true interactions between Cpne4 or its vWA domain and other retinal proteins, the experimental conditions may mimic different states of either Cpne4, or its targets under the two circumstances (protein domain library expressed in yeast cellsversus full retina extract from mouse). Since Copines are majorly associated with membranes, it is possible that lipid membranes play a role in the pull-down assays, even under the mild detergent conditions used for retina lysis. Furthermore, the nature of Y2H libraries, consisting of protein fragments, may reveal epitopes that interact only in certain circumstances with their binding partners. Studies in Dictyostelium point to interactions of CopineA with filamentous but not monomeric actin [24]. This may mean that the protein interactions we recovered could also be affected by the cellular context (cell cortex–actin filaments, plasma membrane, posttranslational modifications, etc.) in addition to the presence or absence of plasma membrane in immediate vicinity.

## 5. Conclusions

To summarize, Cpne4 over-expression leads to morphological changes in the retina. Cpne4 interacts with multiple proteins and is involved in several different pathways within the cells. This study points to several interesting directions, in relation to possible function of Cpne4 in the retina and the rest of the nervous system. Each specific interaction needs to be explored further to establish their roles in development and functioning of the retina.

## Supporting information

S1 Fig HEK293 transfection. Representative images showing full filed views of HEK293 cells transfected with expression constructs for full length Cpne4 (left panel), vWA domain construct (middle panel) and C2 domains construct (right panel). The cells were counterstained for eGFP (*green*), HA (*red*), N-terminal or C-terminal Cpne4 (*white*) antibodies and nuclear marker DAPI (*blue*). For Cpne4 transfected cells, the cells with high levels of transfection gave out long processes (*solid arrow*), whereas those with low levels of transfection did not (*arrowheads*). For vWA domain and C2 domains transfected cells most of the cells did not give out any processes (*dashed arrows*). Scale bar: 50 μm.
(TIF)

S2 Fig Varicosity area measurements. (A) Box plots showing the areas of the regular varicosities on eGFP infected controls, regular size varicosities on Cpne4 infected RGCs, all varicosities (regular and large blebs) on Cpne4 infected RGCs and all varicosities on P0 and P15 Cpne4 infected RGCs. (B) Plot showing correlation between arbor area and varicosity area for Cpne4 infected RGCs. (C) Plot showing correlation between arbor area and varicosity area for eGFP control infected RGCs. ***, p< 0.001.
(TIF)

S3 Fig RNA sequencing data. RNA sequencing data showing FPKM (fragments per kilobase of transcripts per million mapped reads) Map1b, Stx1b; 14-3-3 family- Ywhab, Ywhaz, Ywhae, Ywhag, Ywhaq; SV2a; Bicd2; Pitpnm2, Sptbn1 and Mycbp2invisualbrain areas (MTN:medial temporal nucleas, OPN:olivary pretectal nucleas, SC:superiorcoliculus, LGN: Lateral geniculate nucleas), rest of the brain (Brain) Brn3a wild-type (WT) and knockout (KO) RGCs, Brn3b WT and KO RGCs and rest of the retina (Retina). Brain samples were obtained from P3 WT (wildtype) mice and retina samples from P3 or E15 mice. The genotypes for retina RGC samples are: Brn3b WT = Pax6α:Cre; Brn3b^CKOAP/WT^, Brn3b KO = Pax6α:Cre; Brn3b^CKOAP/KO^, Brn3a WT = Pax6α:Cre; Brn3a^CKOAP/WT^, Brn3a KO = Pax6α:Cre; Brn3a^CKOAP/KO^. Values for brain areas represent medians for three samples (LGN, SC), two samples (whole brain) or individual samples pooled from three animals (MTN and PTA). Retina values represent samples obtained from two pooled retinas, while RGC values are medians of two biological replicates each obtained from 6–8 retinas. Data is extracted from Sajgo et al., 2017[10].
(TIF)

S4 Fig Cpne4 was not pulled down by Bicd2. **Pitpnm2 and Sptbn1.** Western blot images of pull down from co-transfected HEK293 cells using Flag antibody show the total lysate (*I*) and the co-immunoprecipitated (*C*) proteins. Cpne4-vWA (*green*; 31kDa) did not pull-down Flag- Sptbn1 (35.1 kDa), Bicd2 (87.4 kDa) orPitpnm2 (40.7 kDa; all appear as *red* bands). Cpne4-vWA was not detected in the pull-down lane (*C*) for Pitpnm2. Lanes labeled as ‘X’ are excluded from this figure.
(TIF)

S5 Fig Graphs for interesting MS proteins. (A) Bar graphs showing log2 fold change as found in MS analysis for 3 different conditions- Cpne4 vs Adom (Cpne4-vWA), Ctrl (control) vs Adom and Ctrl vs Cpne4 for the proteins Cpne4, 14-3-3 family- Ywhaz, Ywhab, Ywhae, Ywhaq and Ywhag. (B) Bar graphs showing log_2_ fold change as found in MS analysis for 3 different conditions- Cpne4 vs Adom, Ctrl vs Adom and Ctrl vs Cpne4 for the proteins Cpne4, SV2a, Map1b and Stx1b (Syntaxin 1b).
(TIF)

S1 Table LC-MS data. Complete LC-MS dataset including the list of enriched proteins (Cpne4 vs control, Adomain vs control or Cpne4 vs Adomain; Sheet 1) and raw data (Sheet 2) containing the PSM (peptide spectrum match) values for each protein for each replicate (n = 3 experiments).
(XLSX)

S2 Table GO analysis data. GO analysis of enriched proteins from LC-MS data listing most probable biological processes (Sheet 1), molecular function (Sheet 2), cellular component (Sheet 3) and significant pathways from PANTHER (Protein Analysis Through Evolutionary Relationships) analysis (Sheet 4).
(XLSX)

S1 Text DNA sequences of interacting regions of Morn2, HCFC1 and Tox3. (DOCX)

S1 Raw images Raw images for western blots and PCR. (PDF)
