# Supplementary figures and images for: Molecular studies into cell biological role of Copine-4 in Retinal Ganglion Cells
Source: PLoS One. 2021 Nov 30;16(11):e0255860. doi: 10.1371/journal.pone.0255860 (PMC8631636; doi:10.1371/journal.pone.0255860)

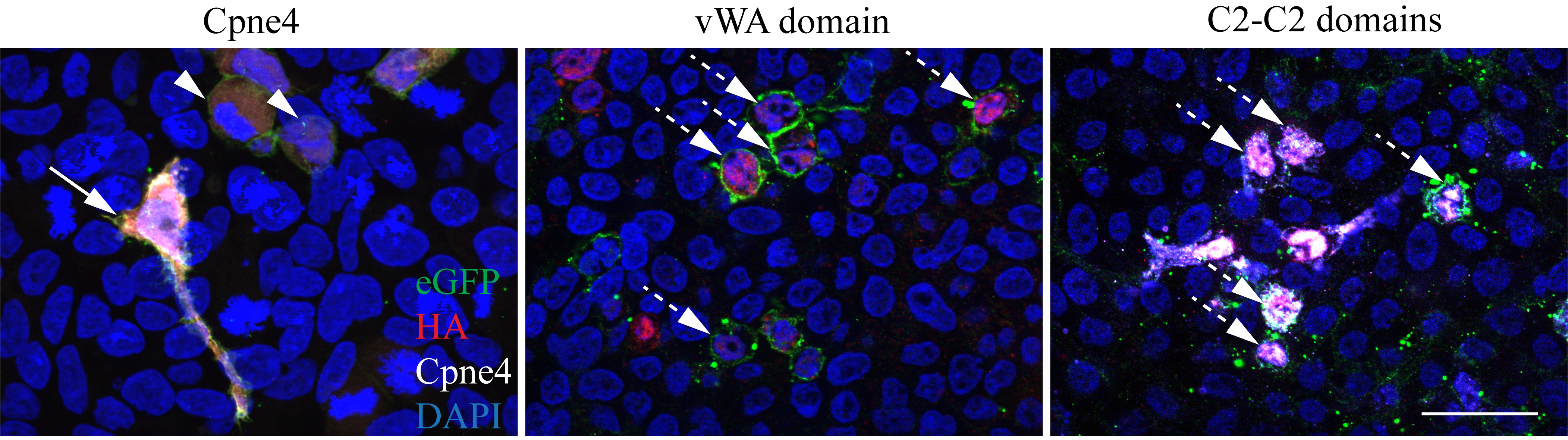

Supplement: S1 Fig — Representative images showing full filed views of HEK293 cells transfected with expression constructs for full length Cpne4 (left panel), vWA domain construct (middle panel) and C2 domains construct (right panel). The cells were counterstained for eGFP (green), HA (red), N-terminal or C-terminal Cpne4 (white) antibodies and nuclear marker DAPI (blue). For Cpne4 transfected cells, the cells with high levels of transfection gave out long processes (solid arrow), whereas those with low levels of transfection did not (arrowheads). For vWA domain and C2 domains transfected cells most of the cells did not give out any processes (dashed arrows). Scale bar: 50 μm. (TIF) [file pone.0255860.s001.tif]

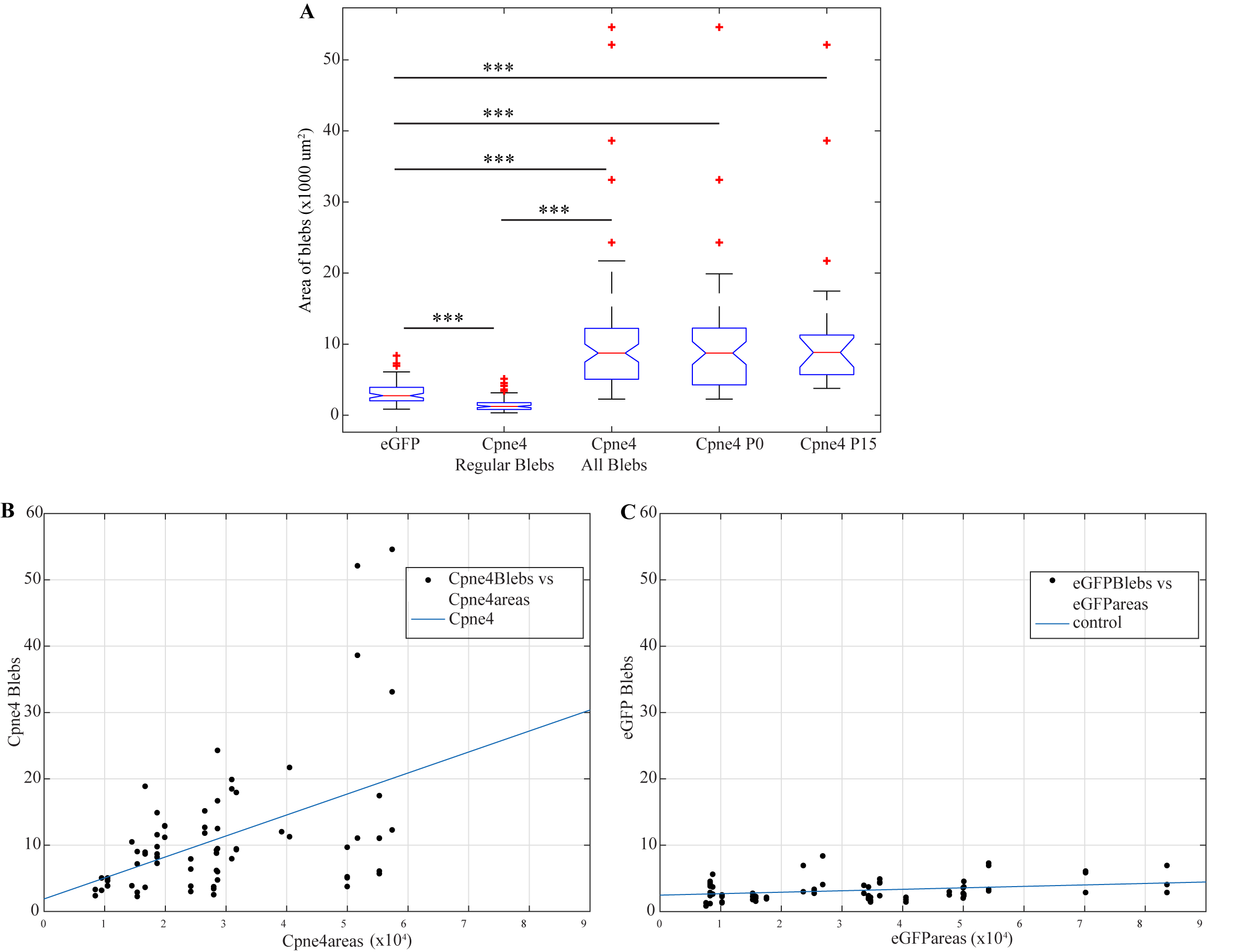

Supplement: S2 Fig — (A) Box plots showing the areas of the regular varicosities on eGFP infected controls, regular size varicosities on Cpne4 infected RGCs, all varicosities (regular and large blebs) on Cpne4 infected RGCs and all varicosities on P0 and P15 Cpne4 infected RGCs. (B) Plot showing correlation between arbor area and varicosity area for Cpne4 infected RGCs. (C) Plot showing correlation between arbor area and varicosity area for eGFP control infected RGCs. ***, p< 0.001. (TIF) [file pone.0255860.s002.tif]

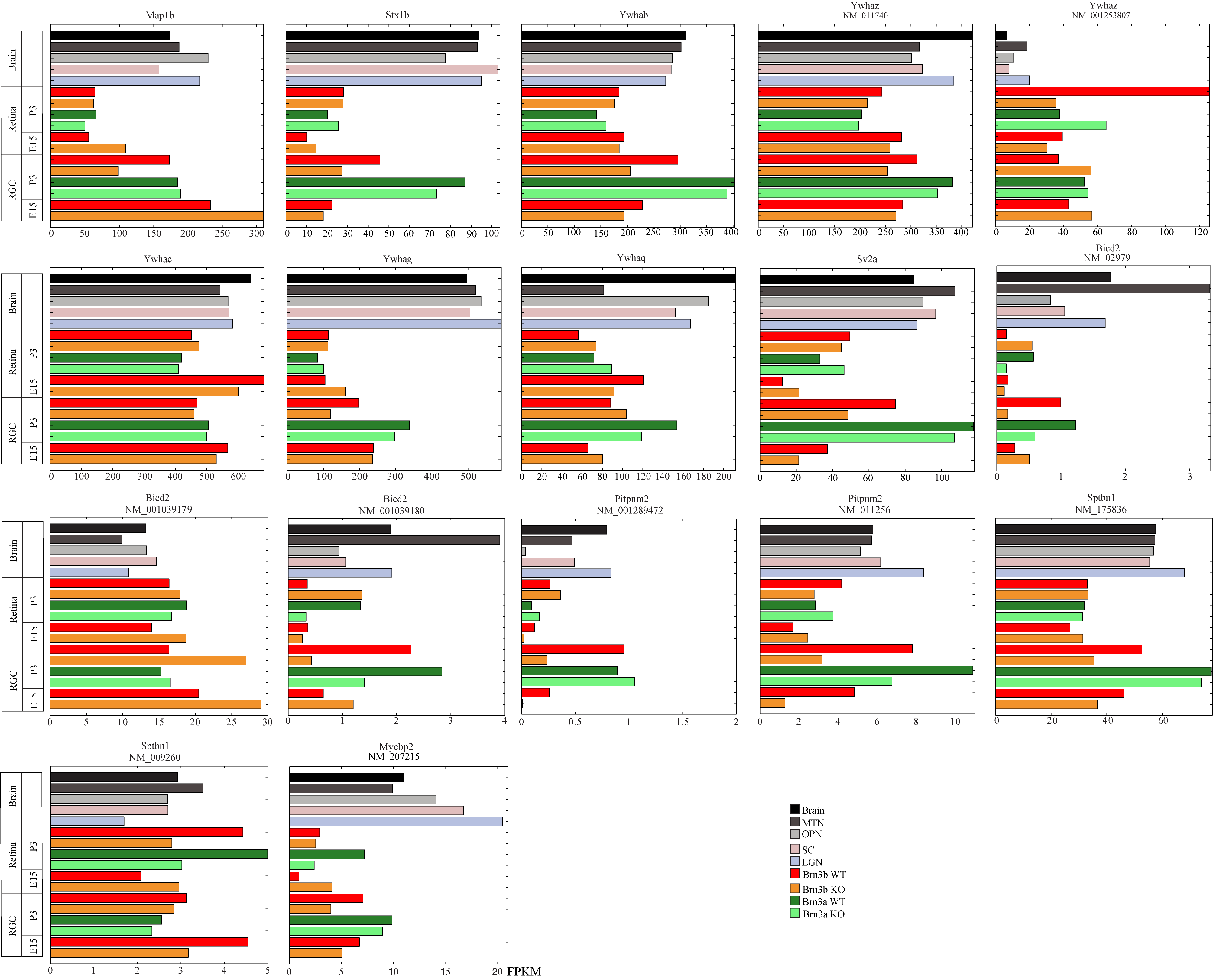

Supplement: S3 Fig — RNA sequencing data showing FPKM (fragments per kilobase of transcripts per million mapped reads) Map1b, Stx1b; 14-3-3 family- Ywhab, Ywhaz, Ywhae, Ywhag, Ywhaq; SV2a; Bicd2; Pitpnm2, Sptbn1 and Mycbp2invisualbrain areas (MTN:medial temporal nucleas, OPN:olivary pretectal nucleas, SC:superiorcoliculus, LGN: Lateral geniculate nucleas), rest of the brain (Brain) Brn3a wild-type (WT) and knockout (KO) RGCs, Brn3b WT and KO RGCs and rest of the retina (Retina). Brain samples were obtained from P3 WT (wildtype) mice and retina samples from P3 or E15 mice. The genotypes for retina RGC samples are: Brn3b WT = Pax6α:Cre; Brn3bCKOAP/WT, Brn3b KO = Pax6α:Cre; Brn3bCKOAP/KO, Brn3a WT = Pax6α:Cre; Brn3aCKOAP/WT, Brn3a KO = Pax6α:Cre; Brn3aCKOAP/KO. Values for brain areas represent medians for three samples (LGN, SC), two samples (whole brain) or individual samples pooled from three animals (MTN and PTA). Retina values represent samples obtained from two pooled retinas, while RGC values are medians of two biological replicates each obtained from 6–8 retinas. Data is extracted from Sajgo et al., 2017[10]. (TIF) [file pone.0255860.s003.tif]

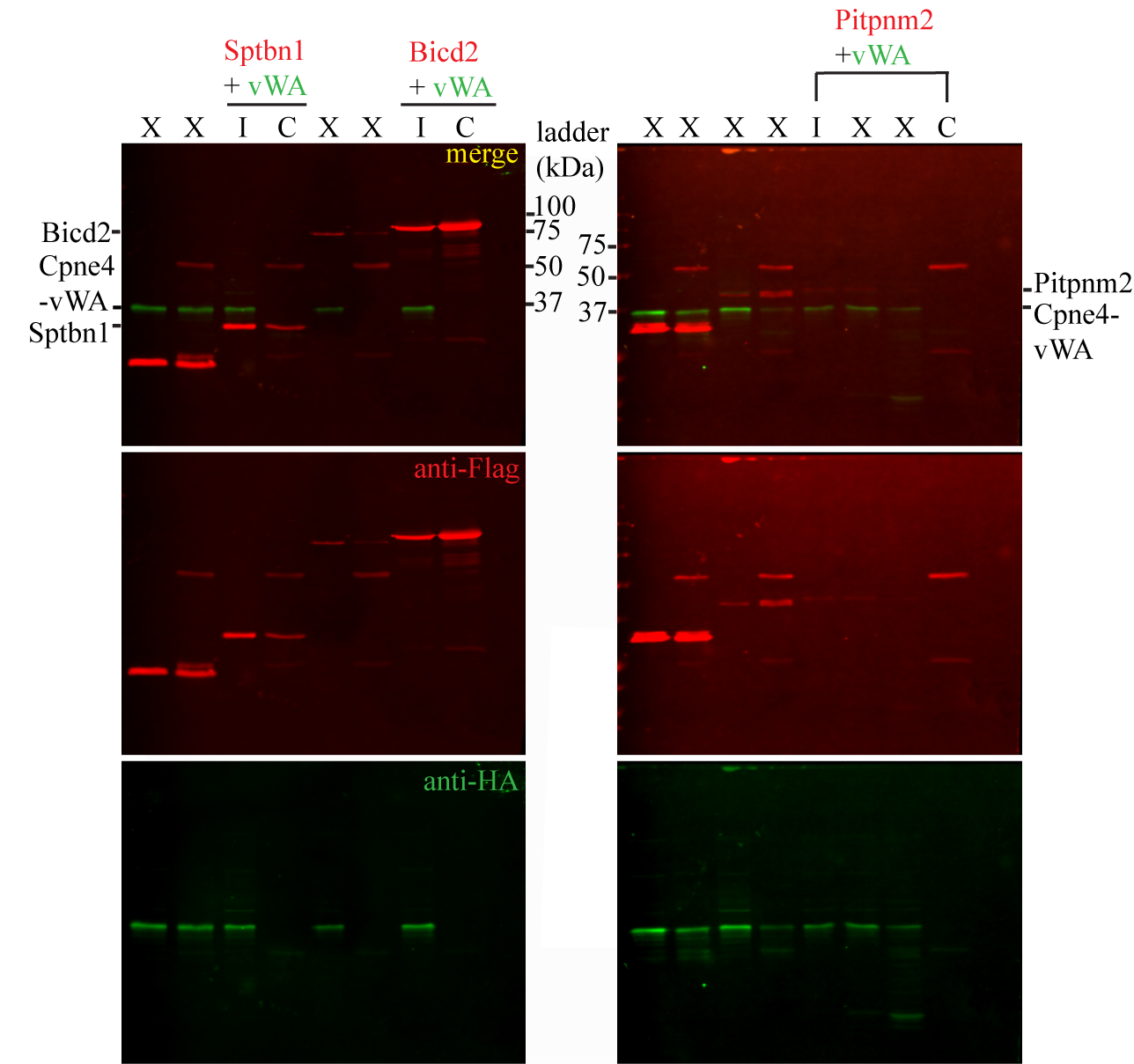

Supplement: S4 Fig — Pitpnm2 and Sptbn1. Western blot images of pull down from co-transfected HEK293 cells using Flag antibody show the total lysate (I) and the co-immunoprecipitated (C) proteins. Cpne4-vWA (green; 31kDa) did not pull-down Flag- Sptbn1 (35.1 kDa), Bicd2 (87.4 kDa) orPitpnm2 (40.7 kDa; all appear as red bands). Cpne4-vWA was not detected in the pull-down lane (C) for Pitpnm2. Lanes labeled as ‘X’ are excluded from this figure. (TIF) [file pone.0255860.s004.tif]

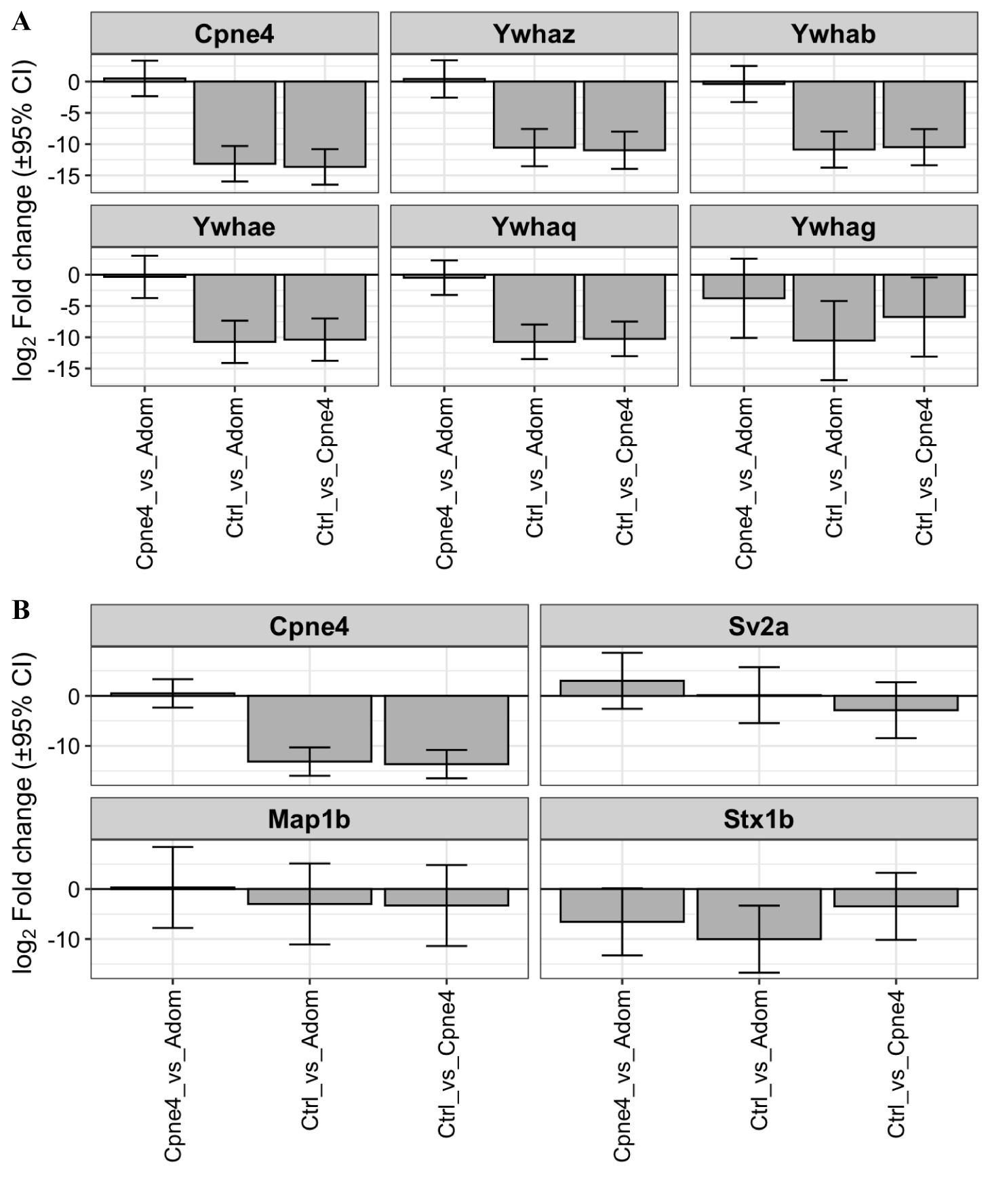

Supplement: S5 Fig — (A) Bar graphs showing log2 fold change as found in MS analysis for 3 different conditions- Cpne4 vs Adom (Cpne4-vWA), Ctrl (control) vs Adom and Ctrl vs Cpne4 for the proteins Cpne4, 14-3-3 family- Ywhaz, Ywhab, Ywhae, Ywhaq and Ywhag. (B) Bar graphs showing log2 fold change as found in MS analysis for 3 different conditions- Cpne4 vs Adom, Ctrl vs Adom and Ctrl vs Cpne4 for the proteins Cpne4, SV2a, Map1b and Stx1b (Syntaxin 1b). (TIF) [file pone.0255860.s005.tif]
