# Supplementary material for: Molecular studies into cell biological role of Copine-4 in Retinal Ganglion Cells
Source: PLoS One. 2021 Nov 30;16(11):e0255860. doi: 10.1371/journal.pone.0255860 (PMC8631636; doi:10.1371/journal.pone.0255860)
Supplement: S1 Text — (DOCX) [file pone.0255860.s008.docx]

**Supplementary DATA**

cDNA sequence of the interactors from the yeast two hybrid interactions-

Morn2:

AAGCAGTGGTATCAACGCAGAGTGGCCATTATGGCCGGGGGAGGCCCGCTCTGTCCTAGAGCCCAGCTCCCTCCAGCCTGCCGAGCTGGCGAGCCTAGGGGAATCGCAGAGTCGCGAAAGCCTGTCCTTCACGTCTCCATCAACTTCGTCAGAAGTATATAAGATCAAATTTATATTTCCAAATGGAGACACATACGATGGCGACTGCACAAGAACTACCTCTGGGATCTGTGAGAGAAACGGGACAGGCACGCACACCACGCCGAACGGGATTGTCTACACAGGAAGCTGGAAAGATGACAAGATGAATGGCTTTGGAAGACTTGAACATTTTTCGGGCGCTGTGTATGAAGGACAGTTTAAGGACAACATGTTTCATGGACTGGGGACTTACACATTCCCAACTGGGGCAAAGTACACTGGAAATTTCAATGAAAATAGGGTAGAAGGTGAGGGAGAATACACTGACACCCAAGGCCTGCAGTGGTGTGGTAACTTCCACTTCACAGCTGCCCCCGGCCTGAAACTAAAGCTCTACATGTAGACCTGCTGCCTTAACGCTGAGATGTGGCCTCTGCAACCCCCCTTAGGCAAAGCAACTGAACCTTCTGCTAAAGTGACCTGCCCTCTTCCGTAAGTCCAATAAAGTTGTCATGCACCCACACCTTTTTGAATTATGTATTGTATGTGTGTGAGCTCATGTGTGTTTATGTGCATGTGTGTGCACATGTGCTTGTATGCATGTGTGTGTGTGTGTCTGTGTGTGAGTACTTGTTCATGCAGGTACATGTGGAAGGCAGAAGTTGATAATAAAATGCCTTTTTCAGGCTAGAACATGCTTTTAATCTCAGCACTCAGGAGAGGCAGGCAGATCTCCTGAGTTGCAGGACAGCCATGGCTACACATCTTGAAAAAGAAAAAAAAAATCCTTTTCAATCCCTTTTTACTTTTTTTTTTTTTTAAAGACATGGTCTTACTATATGGCCATGGTTGGCCTAAAACTCACTGTGTAGACCAGGTTGTCCTCAAACTCACAAAGATCTACCTGTTTCTTCTTACCAAGTGCTAGGATTAAAGGTGTTTGGTCACTAGATTCAATCTTTTCACCTCATTTGTTCCTCAGTGAACCTGGAGCTCACTGTTTCTGCTAATTTGGCTGGCCAGTGGCCCCCAGGAAACGCCCATCTCTGCTCACCCCCAGCTGAGCACGGAGGTTAGGCACGTACCAGCGCGCCTGGAGAGGGAATCAGGGTCTTCACATTTGCAGGGAAAGCACTTTGCCCACTAAGGCATTTCCCCGACTCTTGAATTGTATCTTCATTAGGAAATGACTTTAAATAAATTGTGTGAATCAATTCAGAGTTTATGTATGTTTTAAAATTGGAGCAGTACGGTCCGAATGGTGGCTAAGTCACTATCTAACTCTGCACCTCAACTTTTGTCAGCCCCCATGTCGGCCGCCTCGGCCTCTAGA

HCFC1:

AAGCAGTGGTATCAACGCAGAGTGGCCATTATGGCCGGGGCGAGACACTCGTCGTACCACTAACACCCCCACTGTAGTGCGGATCACTGTGGCTCCTGGGGCATTGGAGAGAGTCCAGGGTACCGTGAAGCCTCAGTGCCAAACCCAGCAGACCAACATGACCACCACCACCATGACTGTGCAGGCCACTGGAGCTCCATGCTCAGCTGGCCCCCTGCTTAGGCCAAGTGTGGCACTGGAGTCTGGGAGCCACAGCCCTGCCTTTGTGCAACTAGCCCTTCCAAGTGTCAGAGTTGGGCTAAGTGGCCCCAGCAGCAAGGACATGCCCACAGGGCGCCAACCAGAGACATATCATACTTACACAACTAATACCCCCATGTCGGCCGCCTCGGCCTCTAGAATCCCGGCGCATGTCGGCCGCCTCGGCCTCTAGA

Tox3:

AAGCAGTGGTATCAACGCAGAGTGGCCATTATGGCCGGGACCTCCAGCAAGCAAATCAGCCACTCCCTCTCCTTCCAGCTCTATCAACGAAGAGGATGCTGATGATGCAAACAGAGCCATTGGAGAGAAAAGAACAGCCCCAGATTCTGGCAAGAAGCCCAAGACTCCAAAGAAAAAGAAAAAGAAAGATCCCAACGAGCCTCAGAAGCCAGTGTCAGCATATGCCCTGTTTTTCAGAGATACACAGGCTGCAATTAAGGGTCAAAACCCCAACGCAACCTTCGGAGAAGTCTCGAAAATCGTAGCATCTATGTGGGACAGCCTTGGGGAGGAGCAAAAGCAGGTATATAAAAGGAAAACAGAAGCTGCCAAGAAAGAATACTTGAAGGCCCTGGCTGCCTACCGGGCTAGCCTCGTTTCCAAGGCTGCTGCTGAATCTGCAGAAGCCCAGACCATCCGTTCTGTCCAGCAGACTCTGGCATCAACCAACCTGACATCCTCCCTCCTCCTGAACACATCACTGTCTCAACATGGGACAGTCCCAGCCTCACCTCAGACTCTTCCACAGTCACTCCCTAGGTCAATTGCCCCCAAACCCTTAACCATGAGACTACCCATGAGCCAGATCGTCACATCAGTCACCATTGCAGCCAACATGCCCTCGAACATTGGGGCTCCACTGATAAGTTCCATGGGGACGACCATGGTTGGTTCAGCAACCTCCACCCAGGTGAGCCCTTCGGTGCAAACCCAGCAGCATCAGATGCAGTTGCAGCAGCAACAGCAGCAGCAGCAGCAGATGCAGCAGATGCAGCAGCAGCAGTTACAGCAGCACCAAATGCATCAGCAGATCCAGCAGCAGATGCAGCAGCAGCATTTTCAGCATCACATGCAGCAGCACCTGCAGCAGCAGCATGTCGGCCGCCTCGGCCTCTAGA
