# Supplementary material for: Molecular studies into cell biological role of Copine-4 in Retinal Ganglion Cells
Source: PLoS One. 2021 Nov 30;16(11):e0255860. doi: 10.1371/journal.pone.0255860 (PMC8631636; doi:10.1371/journal.pone.0255860)
Supplement: S1 Raw images — (PDF) [file pone.0255860.s009.pdf]

Figure 3B- raw image

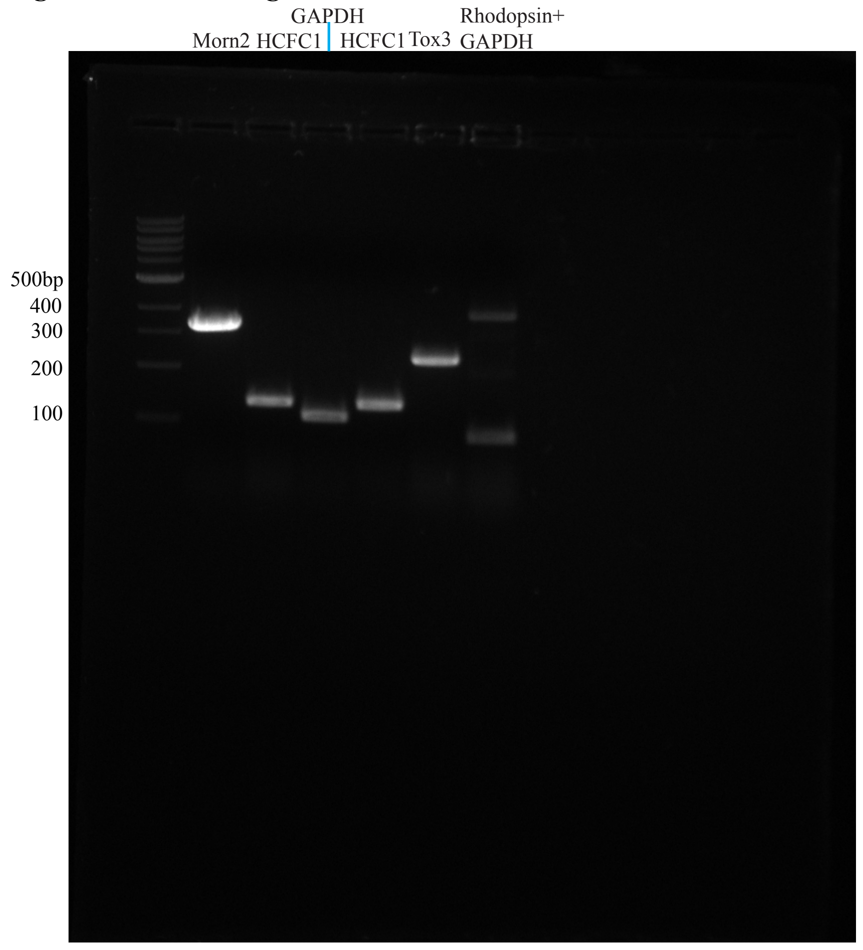

Figure 4H- raw image

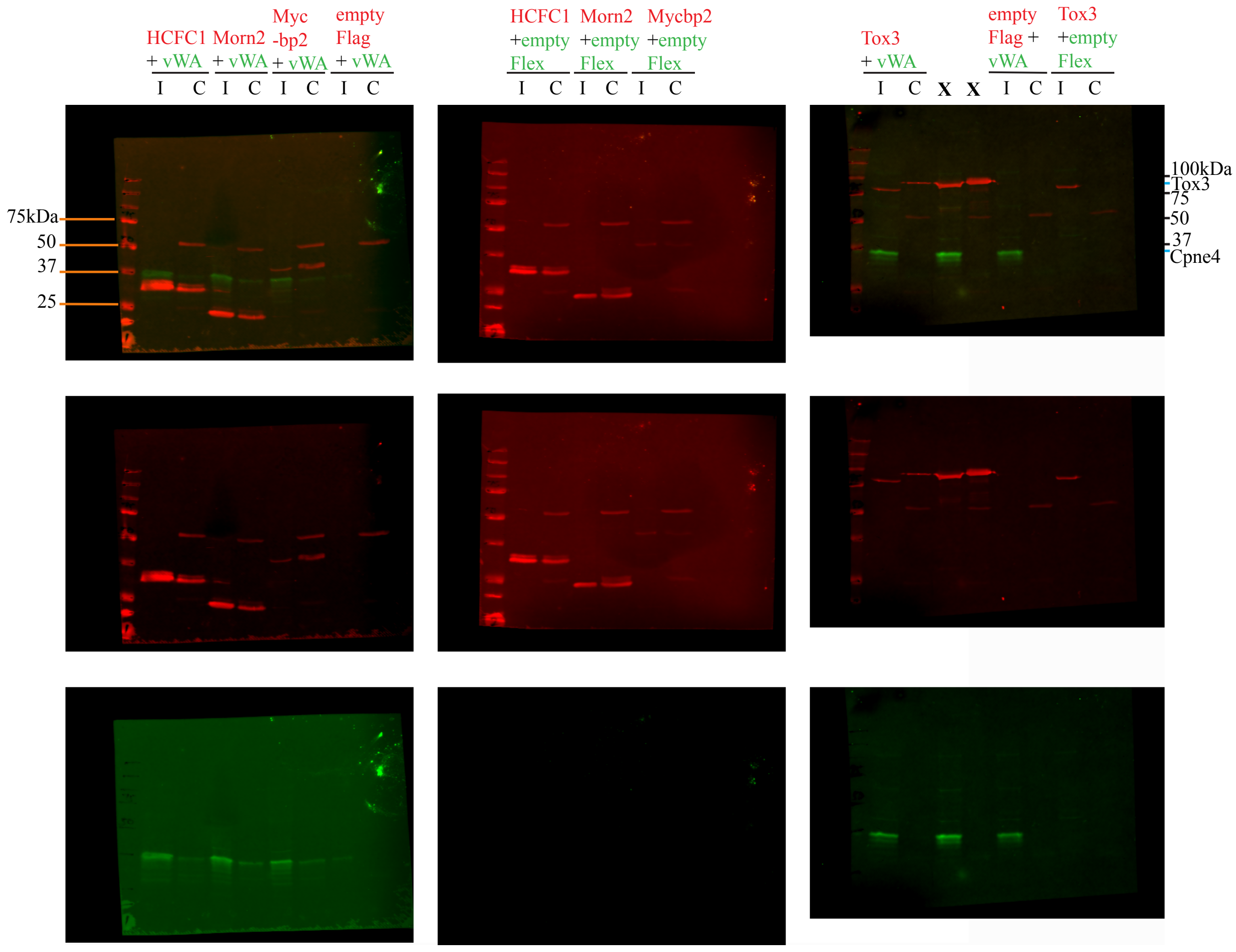

X= lane not used in main figure; see Figure 4 for other lane labels

Figure 6A- raw image

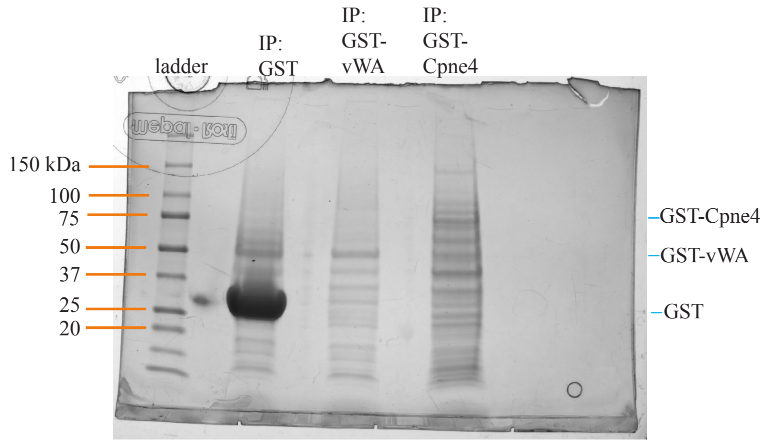

Figure 6B- raw image

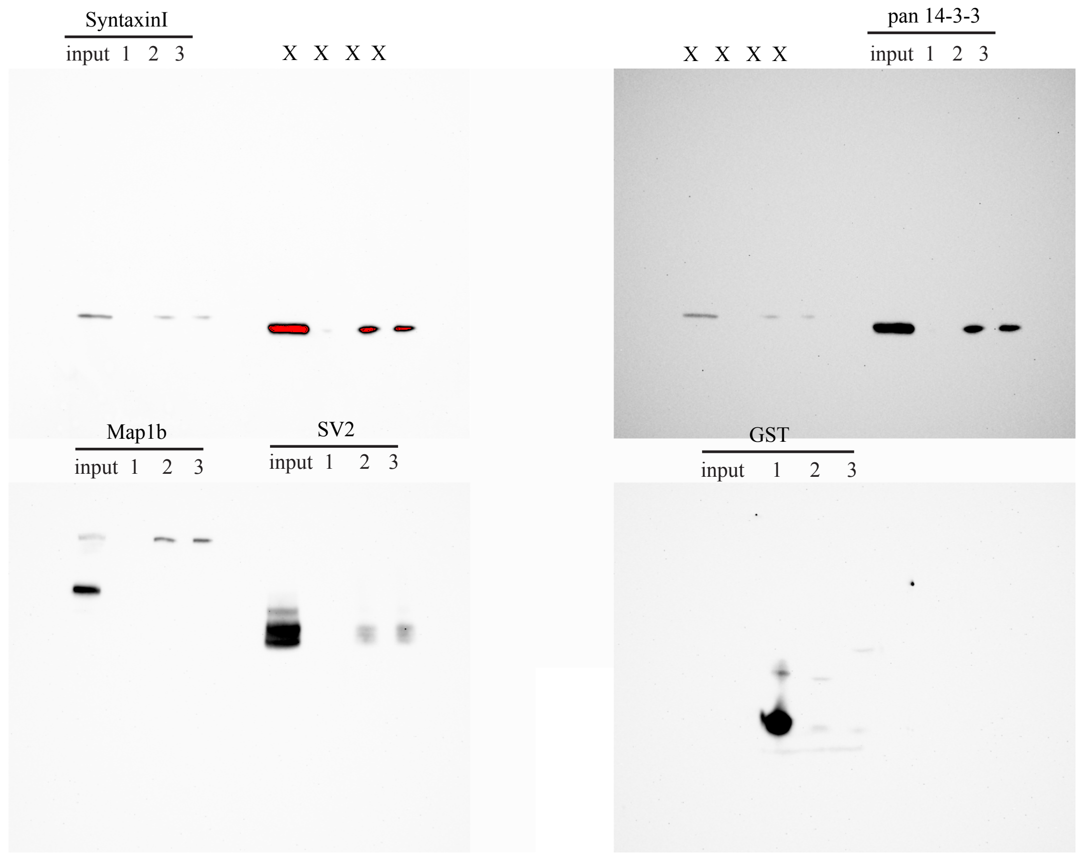

X= lane not used in main figure; see Figure 6 for other lane labels
